# Taxonomic History and State of Knowledge of the Marine Species in Nostocales (Cyanoprokaryote) From the Mexican Atlantic

**DOI:** 10.1002/ece3.71826

**Published:** 2025-07-30

**Authors:** Ernesto Cabrera‐Becerril, Annie May Ek García‐García, María Luisa Núñez Resendiz, Kurt M. Dreckmann, Abel Sentíes

**Affiliations:** ^1^ Doctorado en Ciencias Biológicas y de la Salud Universidad Autónoma Metropolitana Ciudad de México Ciudad de México Mexico; ^2^ Departamento de Hidrobiología Universidad Autónoma Metropolitana‐Iztapalapa (UAM‐I) Ciudad de México Mexico; ^3^ Maestría en Biología Universidad Autónoma Metropolitana‐Iztapalapa (UAM‐I) Ciudad de México Mexico

**Keywords:** classification, diversity, marine phycoflora, taxonomic, taxonomic status

## Abstract

Cyanoprokaryotes are organisms that perform oxygenic photosynthesis. One of the most diverse orders is Nostocales, whose species are characterized by the presence of heterocytes. Phycofloristic lists in the Gulf of Mexico and the Mexican Caribbean have secondarily recorded cyanoprokaryotes. Particularly in Mexico, 1123 species have been recorded, of which only 164 correspond to marine environments. An exhaustive review of the literature on the order Nostocales from the Gulf of Mexico and the Mexican Caribbean was carried out. The list of species was updated taxonomically. Furthermore, the historical analysis of their taxonomy was carried out by consulting the literature corresponding to the starting point established in Bornet and Flahault (1886–1888), as well as previous and later works to date. We found 22 works that include reports of marine Nostocales; 18 correspond to articles and four to book chapters; others contain records presented in a congress meeting. Our updated list of Nostocales from the Mexican Atlantic presents 45 currently taxonomically valid species, distributed in 19 genera and eight families. The states that present the greatest specific richness are Veracruz (33), followed by Quintana Roo (23), Yucatán (18), Campeche (5), Tabasco (4), and Tamaulipas (2). Several attempts at ordering have occurred throughout its history; in the work of Bornet and Flahault, one of the first monographs and compendiums of the group, the order was considered a family, composed of 163 species in 30 genera, 19 sections, two subgenera of seven subtribes, five tribes, one subfamily, one family, and one suborder. Despite these advances, in the last 20 years, Mexican benthic marine cyanoprokaryota species remain little explored, so it is important to continue their registration and analysis.

## Graphical abstract

In this manuscript we contribute a revision of Cyanoprokaryotes Nostocales in the Gulf of Mexico and the Mexican Caribbean; the list of species was updated taxonomically. Also, the historical analysis of their taxonomy was carried out.

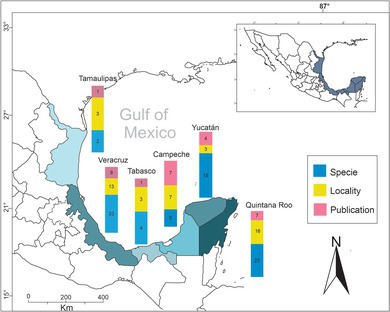

## 1 Introduction

Cyanoprokaryotes are organisms that carry out oxygenic photosynthesis; they have chlorophyll *a* and phycobilins (phycocyanin and phycoerythrin) as main pigments (Komárek 2013). The cell wall of cyanoprokaryotes is composed of an inner cytoplasmic membrane, an outer cytoplasmic membrane, a layer of peptidoglycan between the two membranes, a so‐called S‐layer with a net‐like appearance (two crystalline arrangements of protein subunits), and an outer layer attached to the S‐layer called the sheath (composed of exopolysaccharide fibers) (Hoiczyk 1998; Šmarda et al. 2002). They present different levels of organization ranging from unicellular to thallose, filamentous, or colonial forms, with cellular differentiation (León‐Tejera et al. 2009; Komárek and Anagnostidis 2005; Komárek 2010a, 2010b; Komárek et al. 2014). They can be found throughout the world, in a wide range of environments, both terrestrial and aquatic, in rivers, lakes, seas, coastal lagoons, extreme environments (such as hydrothermal vents and high sulfur waters), hypersaline environments, associated with rocks or other organisms (epizoic and epiphytic, mainly red algae, sea grass beads, or mangrove roots), forming mats resistant to desiccation, and tolerant to changes in salinity (Hoffmann 1999; Whitton and Potts 2012; Hernández‐Casas et al. 2024; Nava‐Olvera et al. 2024). Many species are also harmful algal bloom formers (HABs), which are often potentially toxic (Lind et al. 2016; Poot‐Delgado et al. 2017; Komárek 2010a). Among the most diverse orders, Nostocales Cavalier‐Smith (2002) stands out, made up of 19 families, 128 genera, and 334 species (Hauer and Komárek 2024).

These species differ from the rest by containing heterocytes, whose function is to produce the enzyme nitrogenase (responsible for nitrogen fixation), and akinetes, long‐lasting resistance structures that arise in times of environmental stress (such as times of drought, low temperatures, floods, among others) and have the same metabolic processes as vegetative cells (Komárek 2010a, 2013). Evolutionarily, they represent one of the most recent orders within the group, with the emergence of the heterocyte being an early occurrence within the clade that few genera have specifically lost, as in the case of *Raphidiopsis* F.E. Fritsch & M.F. Rich (Komárek 2013; Strunecký et al. 2023).

In Mexico, specifically in the Gulf of Mexico and the Mexican Caribbean, cyanoprokaryotes have been recorded secondarily, as part of general phycofloristic lists, so there are few works that deal with the group or order and, even less, those that provide morphological descriptions for the identification of the species (Soto et al. 2004; Martínez‐Arroyo et al. 2000; Herrera‐Silveira 2006; Ortegón‐Aznar et al. 2008; Poot‐Delgado and Guzmán Noz 2009; Chuc‐Contreras et al. 2012; del Carmen Merino‐Virgilio et al. 2013; Mateo‐Cid, Mendoza‐González, Ávila Ortiz, and Díaz Martínez 2013; Mateo‐Cid, Mendoza‐González, and Fredericq 2013; González‐Reséndiz et al. 2014; Mendoza‐González et al. 2016). The diversity of Mexican species in marine environments has been little studied; consequently, the lack of studies on the group has generated gaps in the real knowledge of the specific richness in these environments.

In light of the above, this review aims to provide an updated assessment of the current state of knowledge that has not been thoroughly reviewed in recent years and to present a historical overview of the group's taxonomy, including some persistent historical taxonomic issues related to the benthic marine species of Nostocales found in the Mexican Atlantic.

## 2 Taxonomy of Cyanoprokaryotes

The traditional taxonomy of cyanoprokaryotes has been built from the morphology of the species, which is why, throughout history, it has undergone different revisions that have changed both the number of species and higher taxa as observation tools improved. Several attempts at ordering them have occurred throughout their history; the work of Bornet and Flahault (1886/1888), constitutes one of the first monographs and compendiums of the group. In Gomont (1892), the species were classified only as heterocytous and filamentous, presenting 1300 species distributed in 145 genera, 20 families, and three orders, mainly from temperate environments (marine, freshwater, and wet soil). Bornet and Flahault (1886/1888) (Annex 1 in the Supporting Information) are considered the “starting point” in classification; this is the reference point from which the origins of the names and taxonomic classifications of the species within the Nostocales cyanoprokaryotes are derived, and Gomont (1892) of the Oscillatoriales, according to the ICBN in article 13 (Ortega et al. 2001; McNeill et al. 2006 (Vienna Code):27; Guiry and Guiry 2025; Pedroche 2022).

Geitler (1925) divided the cyanoprokaryotes into seven orders: Chroococcales, Entophysalidales, Pleurocapsales, Dermocarpales, Sinphonematales, Nostocales, and Stigonematales; in this work, he grouped taxa registered and created by Gomont (1892), Borzi (1914), and Schaffner (1922). Later, Geitler (1932) adopted Fremy (1939) system and included only three orders, Chroococcales, Chamaesiphonales, and Hormogonales, in addition to suggesting changes to the system recognizing Chroococcales, Pleurocapsales, Dermocarpales, and Hormogonales.

In Fritsch (1945a), species were classified according to their growth form and the presence and absence of certain forms of cellular division and reproductive structures (e.g., Nanocytes), into five orders: Chroococcales, Chamaesiphonales, Pleurocapsales, Nostocales, and Stigonematales.

Until the revision of Stigonematales by Desikachary (1959), the unbranching filamentous cyanoprokaryotes had been placed in the order Nostocales (Komárek et al. 2014); this classification remained stable for almost 24 years. In Smith (1950), all cyanoprokaryotes are grouped into three orders based on the production of exospores, endospores, and the lack of these; these orders are Chamaesiphonales, Chroococcales, and Oscillatoriales.

The works of Drouet (1968, 1973, 1978, 1981), Drouet and Daily (1956) (Annex 2 in the Supporting Information) affected the organization of the order in which revisions were made on the taxonomy of the group, starting from the idea that the majority of the species described at the time were morphological variations (ecophenes: morphological changes as a response to the environmental changes that the species suffer) of a few genotypes. Before this new arrangement, the list of taxonomically accepted species of cyanoprokaryotes corresponded to approximately 2000 species. From this list, Drouet and Daily (1956) rearranged them into only 62 species, which corresponded to two orders: Chroococcales (all coccoid forms) reduced to 17 species from three families and Hormogonales (all filamentous ones) reduced to 26 species. The reason for these reductions was due to the authors assumption that all species corresponded only to a morphological variation (depending on the environment) of certain species, converting many morphologically similar species into single species.

Cyanoprokaryotes had been described using the International Code of Botanical Nomenclature (ICNB previously mentioned), currently recognized as the International Code of Nomenclature for Algae, Fungi, and Plants (ICN or ICNafp), due to their close nature to algae (both, perform oxygenic photosynthesis, share pigments, and are phylogenetically related, as cyanoprokaryotes are ancestors of red and green algae), using botanical criteria for their classification.

The first publications that promote the classification of cyanoprokaryotes with the bacterial classification system are Stanier et al. (1978) and Rippka et al. (1979), who use morphological, physiological, and cytological characteristics as identification criteria in axenic cultures for the classification of cyanoprokaryotes genera and remove the specific epithet, replacing it with a culture number. The classification of Rippka et al. (1979) (Annex 3 in the Supporting Information), reviewed by Castenholz et al. (2001) (Annex 4 in the Supporting Information), included five sections: I. unicellular; II. Colonial; III. simple filaments, which reproduce by breaking the trichome; IV. filaments uniseriate, *heterocytous*, with false branching, which reproduce by hormogonia; and V. filaments with heterocyte*s*, multiseriate, with true branching that reproduce by hormogonia and can develop akinetes.

Nonetheless, while most of the structure of the classification system of Rippka et al. (1979) reviewed by Castenholz et al. (2001) was maintained, the use of molecular techniques utilizing 16S gene sequences and the advance in the study of cyanoprokaryotes with the botanical classification system, used and revised by Komárek and Anagnostidis (since the beginning of the decade of the 90s), left aside the use of this classification system, although some genera and, recently, a phylum Cyanobacteriota continue to be added to the system of Rippka et al. (1979) reviewed by Castenholz et al. (2001) (Oren et al. 2022).

Cavalier‐Smith (2002) elaborated a classification of bacteria including cyanoprokaryotes, dividing the kingdom into two subkingdoms, Negibacteria and Unibacteria; within Negibacteria is the Glyobacteria infrakingdom and within the Cyanoprokaryote division. On the contrary, Anagnostidis and Komárek (1988, 1990) separate cyanoprokaryotes into four orders: Nostocales, Stigonematales, Oscillatoriales, and Chroococcales, using morphology and transmission electron microscopy within the botanical nomenclature code, continuing with the arrangement of Rippka et al. (1979).

Along with the advancement of the bacterial classification system applied to cyanoprokaryotes, the works of Komárek were among the most important from the 1990s onward, as they continued the work of classifying and analyzing the taxonomic position and phylogenetic relationships of bacterial species, incorporating a polyphasic approach to the botanical nomenclature system. In their first compilation work on the former order Chroococcales (which included all the coccale forms described at the time of its publication), Komárek and Anagnostidis (1999) used cellular morphology, the patterns of cell division, and the morphology of the thylakoids (characters observed thanks to transmission electron microscopy) as classification criteria.

Years later, Komárek and Anagnostidis (2005) utilized thylakoid morphology, together with filament apices, and integrated phylogenetic relationship data of the group to build a book that provided a classification scheme for what was then regarded as the Oscillatoriales order. In 2013, Komárek presented a compilation like those of the other orders, using molecular tools, morphology, ecological information, and the nature of the life cycles of the Nostocales. In this compilation, the structure of the order was proposed. However, this publication and the first works, traditionally used, such as Geitler (1932) and Desikachary (1959), are focused on European populations from mainly freshwater environments and temperate climates, leaving aside populations in tropical or extreme environments (Hoffmann et al. 2005).

Over 30 years of work, several modifications to the classification system were developed, following the popularization of the use of molecular tools, especially the 16S gene and the 16S–23S ITS, which have provided the largest number of molecular sequences to the present. Hoffmann et al. (2005) (Annex 5 in the Supporting Information) recognize that up to that point, the taxonomic revisions and important nomenclatural changes have been included in the aforementioned publications, as well as others by the same authors (Komárek and Anagnostidis 1986, 1989; Anagnostidis and Komárek 1985, 1988, 1990). From the studies and modified systems of these authors, several aspects of cyanoprokaryotes are recognized: (a) that it is a clearly polyphyletic group; (b) that the filamentous forms (Oscillatoriales) as well as the coccales forms (Chroococcales) are dispersed throughout the classification system; (c) the morphological classification did not correspond to the molecular data recorded at the time (Hoffmann et al. 2005); (d) heterocytous forms form a monophyletic group; (e) the orders up to that moment present in the classification systems are polyphyletic within themselves; (f) that although several phylogenetically related groups would correspond in terms of the morphology of the thylakoid arrangement, the previous arrangement of Hormogonales that incorporated the filamentous ones, in general, would not correspond with the information obtained so far, which pointed out that several genera of the coccal and the non‐heterocytous filamentous ones should be together within different orders; (g) that the separation between the filamentous group Lyngbyeae (With sheath) and Vaginarieae (Without sheath) proposed by Gomont (1890, 1892) did not correspond with the phylogeny of the group.

Subsequently, the work of Komárek et al. (2014) introduced a new taxonomic arrangement based on taxonomic information generated with molecular data, using the 16S gene, to resolve various taxonomic problems derived from the different studies described. In this way, the subclasses proposed by Hoffmann et al. (2005) are recognized, but the clarification that, depending on the emergence of new information, these levels will change, was made like fragmenting into more subclasses, because Synechococcophycidae and Oscillatoriophycidae are polyphyletic. In this proposed system, the sequences of the 16S ribosomal RNA gene were analyzed, and it was suggested to maintain the categories of information generated in the Heterocytous book by Komárek (2013) and applied it to all orders. In this way, these categories are included as: (a) category 1, all genera that have phylogenetic information and a type specimen; (b) category 2, genera with molecular information without a type specimen; (c) category 3, those recognized as morphogenes requiring revision, which include phylogenetic information but are polyphyletic or paraphyletic, with no registered type species; (d) category 4, those genera whose discovery occurred a long time ago, but which, due to their nature had not been able to be cultivated and do not have molecular information; (e) category 5, those genera that are taxonomically invalid, although some recent one with phylogenetic information, do not comply with the requirements to be recognized as valid in either of the two taxonomic nomenclature codes approved for the group (International Nomenclatural Code of Bacteria and the International Nomenclatural Code of Algae, Fungi and Plants). It is worth highlighting that, from this moment on, new orders have been recognized, including Spirulinales, Pleurocapsales, and Chroococcidiopsidales, changing the status of several genera and families.

In recent years, Hauer and Komárek (2024) (Annex 7 in the Supporting Information), together with the University of Bohemia and the Institute of Botany of the Academy of Sciences of the Czech Republic, generated the online database of cyanoprokaryota genera (CyanoDB‐2), considering which the most updated database of orders, genera, and some of the recognized species until before 2023. It was based on the latest work by Komárek et al. (2014), and, from the moment of its creation, it was updated with the works and descriptions of the main species and genera until April 2023. This database contains 2278 taxa considered valid from the phylum, including 451 genera and 1724 species.

In Strunecký et al. (2023), the most recent review of the entire group of cyanoprokaryotes, the most recent information was incorporated into the classification system of cyanoprokaryotes, based on the analysis of current sequences of the 16S gene, incorporating new taxa that, until now, have not been properly placed in the pre‐established orders in 2014. It is worth highlighting the addition to the classification system, the non‐photosynthetic Cyanoprokaryote, of the new class Vampirovibriophyceae. These organisms are phylogenetically related to cyanoprokaryotes lacking the photosynthetic machinery, and they have been described from samples of digestive tracts in insects and mammals, including humans (Strunecký et al. 2023; Hu and Rzymski 2022). For the latter, class II., corresponding in this revision to Cyanophyceae, is composed of 19 orders, of which 10 orders were added (Aegeococcocales, Acaryochloridales, Prochlorotrichales, Nodosilineales, Oculatellales, Gomontiellales, Leptolyngbyales, Geitlerinematales, Desertifilales, and Coleofasciculales), as well as 15 new families (Lusitaniellaceae, Wilmottiaceae, Aerosakkonemataceae, Neosynechococaceae, Konicacronemataceae, Persinemataceae, Halothecaceae, Cymatolegaceae, Nodosilineaceae, Thalassoporaceae, Aegeococcaceae, and Anthocerotibacteraceae).

### 2.1 Taxonomic History of the Order Nostocales

The first publication in which Nostocales appeared recognized as an order was the Bornet and Flahault (1886/1888), in which they classified the order Nostocales as Ordo (order) I, Schizophyceae. This designation corresponds to the family category in contemporary taxonomic nomenclature (Dreckmann and De Lara‐Isassi 2001), composed of 163 species, 30 genera, in 19 sections, which include 2 subgenera, 7 subtribes, 5 tribes, a subfamily, a family, and a suborder (Annex 1 in the Supporting Information).

In the 1940s, Fritsch (1945a, 1945b) described the order found within the Nostocales and Stigonematales. In Nostocales, all non‐heterotric filamentous forms (those not exhibiting erect thalli growth from prostrate thalli) were categorized; however, considering the facultative presence of the heterocyte and the presence of akinetes (as a characteristic of lesser taxonomic importance), they were grouped together with the Oscillatoriales. In Stigonematales, heterotrichous forms, predominantly featuring heterocyte*s* and true branching, were grouped. Smith (1950) classified Nostocales within the Order Oscillatoriales, characterized by the absence of exospores or endospores, a filamentous morphology with trichomes composed of continuous cells, reproduction via hormogonia and akinetes, and occasionally the presence of heterocytes. Within this order there were six families: Oscillatoriaceae, Rivulariaceae, Nostocaceae, Scytonemataceae, and Stigonemataceae. The presence or absence of the heterocyte primarily distinguished Oscillatoriaceae from the other families.

Species with trichomes without branches, containing heterocytes and akinetes, comprise the Nostocaceae family. Scytonemataceae include filamentous organisms, trichomes with colored “sheaths”, with hormogonia and false branching. Stigonemataceae were separated by the presence of true branching and multiserial trichomes. Rivulariaceae presented heterocyte*s* and attenuated trichomes from the base toward the apex or from the middle of the trichomes towards the apex (Smith 1950; Bold and Wynne 1978).

From the work of Drouet (1968, 1973) (Annex 2 in the Supporting Information), there was a rearrangement of all cyanoprokaryote species into only 62 species. In the work of Drouet (1968), they were classified in a single order with eight families, four of which included the order Nostocales with the families Nostocaceae, Rivulariaceae, Scytonemataceae, and Stigonemataceae. Drouet described the separation between these and the other filamentous families based on the presence of heterocytes. The Nostocaceae family was distinguished by the presence of akinetes and the production of spores; Rivulariaceae, due to its habitat described as “almost completely marine” and also to the shape of the trichome apex, as they have heteropolar trichomes. The species of the Scytonemataceae family were related to *Lyngbya* C. Agardh ex Gomont, differentiated by the branching pattern and the presence of heterocytes. The Stigonemataceae family is characterized by its pseudoparenchymatous, multiserial structure and true branching.

For Drouet (1973), the order Nostocales was considered within the order Hormogonales, with the families Nostocaceae and Stigonemataceae, and it included only nine species in total. Bornet & Flahault (1886–1888) originally described 163 species, but reduced 48 of them to synonyms of only nine species, which they recognized as valid. It should be noted that a good part of the species of the order (35) was rearranged into three species: *Anabaina oscillarioides* Bory ex Bornet & Flahault (reported with this name by the authors), *Calothrix crustacea* Thuret, and *Scytonema hofmannii* C. Agardh.

Rippka et al. (1979) (Annex 3 in the Supporting Information) proposed the use of the bacteriological code. In a first version of the proposal, the order Nostocales was distributed in two of the subsections into which it was divided at that time, called Sections IV and V, described below. Subsequently, Castenholz et al. (2001) (Annex 4 in the Supporting Information) took up the previous proposal and, using the information available up to that point, with molecular tools and the recognition of some species and genera in the code, made a new proposal. According to this work, the current order Nostocales was divided into two subsections, as in the case of the classification of Rippka et al. (1979); however, considering new discoveries, a considerable number of new species have been added, some of which already had a description from an axenic culture. Likewise, the phylogenetic concepts of the *cluster* (grouping or clade) are added, based on the first analyses with molecular tools using the 16S gene of the axenic cultures that generated the classification system; the formation of different clades that are described are recognized as being part of a species.

Section IV, Nostocales was divided into two subsections, IV.1 and IV.2. Section IV.1 includes the genera *Anabaena* Bory ex Bornet & Flahault nom. cons. 1886, *Anabaenopsis* V. V. Miller 1923, *Aphanizomenon* Morren ex Bornet & Flahault 1886, *Cyanospira* Chodat 1921, *Cylindrospermopsis* G. Seenayya & N. Subba Raju 1972, *Cylindrospermum* Kützing ex Bornet & Flahault 1886, and *Nodularia* (Mertens in Jürgens) ex Bornet & Flahault 1886.

Section IV, Nostocales, was divided into two subsections IV.1 and IV.1 and IV. Section IV.1 included the genera *Anabaena* Bory ex Bornet & Flahault nom. cons. 1886, *Anabaenopsis* V.V. Miller 1923, *Aphanizomenon* Morren ex Bornet & Flahault 1883, *Cyanospira* Chodat 1921, *Cylindrospermopsis* G. Seenayya & N. Subba Raju 1972, *Cylindrospermum* Kützing ex Bornet & Flahault 1886, *Nodularia* (Mertens in Jürgens) ex Bornet & Flahault 1886, *Nostoc* Vaucher ex Bornet & Flahault nom. cons. 1886, and *Scytonema*; in this case, all members of Nostocaceae from Komárek and Anagnostidis (1989) were included, although the members of the section were previously divided into two families, Nostocaceae and Anabaenoide. All the genera included within this subdivision essentially coincide in the form of growth, with isopolar trichomes without basal heterocyte*s*. Section IV.2 included species of the genus *Calothrix* Agardh ex Bornet & Flahault 1886 and *Rivularia* (Roth) C. Agardh ex Bornet & Flahault 1942, as well as others with similar growth forms, heteropolar trichomes, with different degrees of attenuation at the apex, and basal and intercalary heterocytes (Castenholz 2015).

At the beginning of the 21st century, Cavalier‐Smith (2002) described Nostocales as an order of the class Hormogoneae, preserving part of the taxonomic arrangement of Drouet (1973), considering Hormogoneae and Oscillatoriales, but changing its position as a class. Anagnostidis and Komárek (1988, 1990) identified two groups of cyanoprokaryotes with specialized cells, Nostocales and Stigonematales, because their branching patterns are different, just like it had been recognized before (Hoffmann et al. 2005). However, the advances made possible by the introduction of molecular tools demonstrated that all heterocytous cyanoprokaryotes form a monophyletic group within the cyanoprokaryotes (Hoffmann et al. 2005) and that the three main groups likely diverged from a common ancestor; this finding aligns with the arrangement of thylakoids typically observed in heterocytous cyanoprokaryotes.

Hoffmann et al. (2005) (Annex 5 in the Supporting Information) presented a review prior to the Nostocales book by Komárek (2013). In their classification, Nostocales are recognized as subclass Nostochophycidae, an order, Nostocales, and 10 families. In this classification, due to the work with the 16S gene, the order Stigonematales (until now separated from Nostocales by the branching pattern) is rearranged and becomes three families of the subclass, as it was observed that members of the genus *Scytonema* (with false branching) were phylogenetically related to members of the order Stigonematales (with true branching), in addition to recognizing that they are two polyphyletic orders.

Komárek (2013) (Annex 6 in the Supporting Information) carried out some of the most important work for the order, compiling information on the species recognized so far, while also relying on the molecular analyses available. Also present are descriptions made of many species, which include illustrations and information on the general biology of the species of the order Nostocales; therefore, this work has constituted the basis for the taxonomic identification of the species in the floristic record.

The classification system of Komárek (2013) was developed using the polyphasic approach that included ecology, morphology, and microscopy; thus, the order was made up of 10 families and 101 genera. On the contrary, we distinguished genera that incorporate molecular information and type species, molecular information without type, species without phylogenetic or molecular information, genera that have problems with the validity of their name, and those that are doubtful. Additionally, each genus described included sections with species that require additional studies. Unfortunately, many tropical species described for Mexican marine environments are usually found in these sections.

In the CyanoDB 2 database by Hauer and Komárek (2024) (Annex 7 in the Supporting Information), various instances of names that have been synonymous or deemed invalid due to their earlier descriptions (prior to the starting point) can be identified. The distinction of the Calotrichaceae family can be highlighted, whose taxonomic position remains in question even though several species of the *Calothrix* genus have been sequenced. Some species have been separated from the genus Nostoc, increasing from 26 to 34 genera included in this family; 10 new families are included compared to the Komárek (2013) system: Aphanizomenonaceae, Calotrichaceae, Capsosiraceae, Cyanomargaritaceae, Dapisostemonaceae, Fortieaceae, Geitleriaceae, Godleyaceae, Heteroscytonemataceae, Tolypotrichaceae, and the Nostochopsidaceae family is eliminated.

Strunecký et al. (2023) (Table 1) presented the most recent revision of the group, in which the Nostocales order has undergone several modifications at the family and generic level. The number of families was reduced from 19 to 14 and 138 genera. Symphyonemataceae, proposed in the system of Hoffmann et al. (2005), is integrated within Scytonemataceae, including some genera that still lack DNA sequences; Heteroscytonemataceae, although it remains a monogeneric family, its phylogenetic position was uncertain, as, although morphologically it would belong to the previous family, the production of saxitoxins separates it from the latter. The Hapalosiphonaceae family has been restructured to include Nostochopsidaceae, Fischerellaceae, and Chlorogloeopsidaceae; representatives of the family include the three branching types T, V, or Y, which were once of taxonomic importance (Komárek 2013). Although there is phylogenetic information for several of the genera, there is a lack of clarity in defining some members of the family; consequently, more comprehensive analyses at the generic level are necessary to reestablish these linkages. The Hapalosiphonaceae family has been restructured to include Nostochopsidaceae, Fischerellaceae, and Chlorogloeopsidaceae; representatives of the family include the three branching types T, V, or Y, which were once of taxonomic importance (Komárek 2013). Although phylogenetic information exists for several genera, the definitions of some genera within the family remain unclear, necessitating more in‐depth analyses at the generic level to clarify these relationships. Geitleriaceae is a family separated from Hapalosiphonaceae by Kilgore et al. (2018). The genera within this family lack heterocyte*s* and all present true branching of all types (“Y”, “T”, and “V” branching). The available phylogenetic information separated the Leptobasaceae family, also known as the Fortieaceae family, which was composed of species of the *Rivularia* or *Calothrix* type, but with intercalary heterocyte*s* and, sometimes, with open filaments at the apex. For the Nostocaceae family, it was suggested that to better define its members and their relationship with the other families, it is necessary to use other regions of the genome that provide greater resolution; therefore, the arrangement of Nostocaceae, Nodulariaceae, and Aphanizomenonaceae will be modified to the extent that the above occurs (Strunecký et al. 2023). The families Gloeotrichiaceae and Cyanomargaritaceae have been added to the Aphanizomenonaceae family, which did not happen previously (Komárek et al. 2014; Hauer and Komárek 2024), although the analysis of newly collected material is required to give greater certainty to this statement (Strunecký et al. 2023). The Rhizonemataceae family is also added to the order, and although its position is recognized, thanks to phylogenetic analyses, a greater amount of material needs to be analyzed to be able to support the new position of this monogeneric family (Strunecký et al. 2023) (Table 1).

**TABLE 1:** Classification system proposed by Strunecký et al. (2023).

Order	Family	Genera	Others
Nostocales	Aphanizomenaceae incl. Gloeotrichiaceae Komárek et al. Cyanomargaritaceae Shalygin et al.).	*Amphiheterocytum* Sant Anna et al.	
		*Anabaena* Bory ex Bornet et Flahault	Before Nostocaceae
		*Aphanizomenon* Morren ex Bornet et Flahault	Before Aphanizomenonaceae
		*Cuspidothrix* Rajaniemi, Komárek, Willame, Hrouzek, Kaštovská, Hoffmann et Sivonen	
		*Cyanomargarita* Shalygin, Shalygina et Johansen	Before Cyanomargaritaceae Shalygin et al., this family disappears in this classification system.
		*Cylindrospermum* Kützing ex Bornet et Flahault	Before Nostocaceae
		*Dolichospermum* (Ralfs ex Bornet et Flahault) Wacklin, Hoffmann et Komárek	Before Geitleriaceae Kilgore et Johansen
		*Gloeotrichia* J. Agardh ex Bornet et Flahault	Before Geitleriaceae Kilgore et Johansen 2018
		*Hydrocoryne* Schwabe ex Bornet et Flahault	Before Nostocaceae
		*Macrospermum* Komárek	Before Nostocaceae
		*Neowollea* Tawong	Before Nostocaceae
		*Raphidiopsis* Fritsch et Rich	
		*Sphaerospermopsis* Zapomělová, Jezberová, Hrouzek, Hisem, Řeháková et Komárková	
		*Trichormus* (Ralfs ex Bornet et Flahault) Komárek et Anagnostidis	Before Nostocaceae
		*Wollea* Bornet et Flahault	Before Nostocaceae
	Capsosiraceae Geitler	*Capsosira* Kützing ex Bornet et Flahault	
		*Desmosiphon* Borzi	
		*Nematoplaca* Geitler	
		*Stauromatonema* Frémy	
	Geitleriaceae (recently separated from Hapalosiphonaceae)	*Geitleria* Friedmann	
	Hapalosiphonaceae (incl. Nostochopsidaceae Geitler, Fischerellaceae Anagnostidis, and Komarek, Chlorogloeopsidaceae Mitra and Pandey).	*Albrightia* Copeland	
		*Baradlaia* Palik	Before Nostocaceae Eichler
		*Brachytrichiopsis* Jao	
		*Chlorogloeopsis* Mitra et Pandey	Before in his own family Chlorogloeopsidaceae Mitra et Pandey
		*Chondrogloea* Schmidle	
		*Compactonostoc* F. Cai et R. Li	Before Nostocaceae
		*Dictyophoron* Komárek, Komárková, Ventura, Kozlíková‐Zapomělová et Rejmánková	
		*Fischerella* (Bornet et Flahault) Gomont	
		*Fischerellopsis* Fritsch	
		*Halotia* Genuarion et al.	Before Nostocaceae
		*Handeliella* Skuja in Handel‐Mazzetti	Before Nostocaceae
		*Hapalosiphon* Nägeli in Kützing ex Bornet et Flahault	
		*Hyphomorpha* Borzi	Before in Capsosiraceae Geitler
		*Komarekiella* G.S. Hentschke, J.R. Johansen et C.L. Sant'anna	Before Nostocaceae
		*Leptopogon* Borzi	


		*Letestuinema* Frémy	Before Capsosiraceae Geitler
		*Loefgrenia* Gomont	
		*Loriella* Borzì	
		*Mastigocladus* Cohn ex Kirchner	
		*Mastigocoleopsis* Geitler	
		*Matteia* Borzì	
		*Mojavia* Řeháková et Johansen	Before Nostocaceae
		*Nostochopsis* Wood ex Bornet et Flahault	
		*Pelatocladus* Johansen et Vaccarino in Miscoe et al.	
		*Reptodigitus* Casamatta, Villanueva, Stocks, Vaccarino et Johansen	
		*Schmidleinema* De Toni	
		*Spelaeopogon* Borzi	
		*Thalpophila* Borzì	
		*Westiella* Borzì	
	*Westiellopsis* Janet	
	Heteroscytonemataceae	*Heteroscytonema* McGregor et Sendal in Sendal & McGregor	Morphologically Scytonemataceae, producer of cyanotoxins
	Leptobasaceae	*Aulosira* Kirchner ex Bornet et Flahault	
		*Calochaete* Hauer, Bohunická & Mühlsteinová	Before in Microchaetaceae Lemmermann
		*Camptylonemopsis* Desikachary	Before en Microchaetaceae Lemmermann
		*Colteronema* Copeland	Without molecular information Before Hapalosiphonaceae Elenkin
		*Fortiea* De Toni	Before in Fortieaceae Komárek et al. (2014)
		*Thiochaete* Welsh	Before Nostocaceae
	Nodulariaceae	*Aliinostoc* Bagchi, Bubey et Singh	Before Nostocaceae Eichler
		*Amazonocrinis* Alvarenga et al.	Before Nostocaceae Eichler
		*Anabaenopsis* (Woloszyńska) Miller	Before Aphanizomenonaceae
		*Atlanticothrix* Alvarenga et al.	Before Nostocaceae Eichler
		*Chrysosporum* Zapomelova, Skácelová, Pumman, Kopp et Janeček	Before Aphanizomenonaceae
		*Cyanocohniella* Kaštovský, Berrendero, Hladil et Johansen	
		*Cyanospira* Florenzano, Sili, Pelosi et Vincenzini	Before Aphanizomenonaceae
		*Desikacharya* Saraf, Dawda et P. Singh	Before Nostocaceae
		*Goleter* Miscoe et Johansen in Miscoe et al.	Before Nostocaceae
		*Minunostoc* F. Cai et R. Li	Before Nostocaceae
		*Nodularia* (Mertens in Jürgens) ex Bornet et Flahault	Before Aphanizomenonaceae
		*Pseudoaliinostoc* Lee, Ki et Lee	Before Nostocaceae
*Purpureonostoc* Cai et Li		Before Nostocaceae
*Umezakia* M.Watanabe	

	Nostocaceae	*Dendronalium* Alvarenga et al.	Before Nostocaceae
		*Desmonostoc* Hrouzek et Ventura	
		*Heterocyanococcus* Kufferath	Before in Chlorogloeopsidaceae Mitra et Pandey
		*Nostoc* Vaucher ex Bornet et Flahault	
		*Parakomarekiella* Soares, Ramos et Portugal	
		*Roholtiella* Bohunická, Pietrasiak et Johansen	
		*Violetonostoc* F. Cai et R. Li	
	Rivulariaceae (Includes Calothricaceae)	*Calothrix* Agardh ex Bornet et Flahault, 1886	Before in Calotrichaceae Bennet et Murray 1889
		*Dichothrix* Zanardini ex Bornet et Flahault	
		*Dulcicalothrix* Saraf, Sudkar, Dawda, Gaysina, Gabidullin, Kumat, Behere, Kotulkar, Batule et Singh	Before in Calotrichaceae Bennet et Murray 1889
		*Gardnerula* De Toni	
		*Isactis* Thuret ex Bornet et Flahault	
		*Kyrtuthrix* Ercegović	
		*Macrochaete* Berrendero, Johansen et Kastovsky in Berrendero et al.	
		*Mastigocoleus* Lagerheim ex Bornet et Flahault	
		*Nunduva* González–Resendiz, León–Tejera et Johansen in González–Resendiz et al.	
		*Phyllonema* Alvarenga et al.	
		*Richelia* J. Schmidt in Ostenfeld et J. Schmidt	Before Nostocaceae
		*Rivularia* [Roth] C. Agardh ex Bornet et Flahault	
		*Sacconema* Borzi ex Bornet et Flahault	
	Rizhonemataceae	*Rhizonema* Lücking & Barrie	
	Scytonemataceae. Include Symphyonemataceae Hoffmann et al. (2005)	*Petalonema* Berkeley ex Correns	
		*Chakia* Komárková, Zapomělová et Komárek	
		*Symphyonema* Jao	
		*Mastigocladopsis* Iyengar et Desikachary	Before in Symphyonemataceae Hoffman et al.
		*Iphinoe* Lamprinou et Pantazidou in Lamprinou et al.	
		*Spelaeonaias* Lamprinou, Christodoulou, Hernández‐Mariné et Economou‐Amilli in Lamprinou et al.	
		*Aetokthonos* Wilde et Johansen in Wilde et al.	Unbranched, Toxic
		*Adrianema* De Toni	Without molecular information, Before Symphyonemataceae Hoffman et al.
		*Croatella* Ercegović	Without molecular information. Before Tolypotrichaceae Hauer, Bohunická, Johansen, Mareš et Berrendero‐Gómez
		*Voukiella* Ercegović	Without molecular information, Before in Symphyonemataceae Hoffman et al.
		Brachytrichia Zanardini ex Bornet et Flahault 1887	Without molecular information, Before Symphyonemataceae Hoffman et al.
*Herpyzonema* Weber Bosse		Without molecular information


		*Iyengariella* Desikachary	Without molecular information. Before in Symphyonemataceae Hoffman et al.
	*Parenchymorpha* Tseng et Hua	Without molecular information
	Stigonemataceae	*Cyanobotrys* L. Hoffman	
		*Doliocatella* Geitler	
		*Homoeoptyche* Skuja	
		*Pulvinularia* Borzi	
		*Stigonema* Agardh ex Bornet et Flahault	
	Tolypotrichaceae (syn. Borzinemataceae Geitler, include. Dapisostemonaceae Hentschke et al. y Godleyaceae Hauer et al.)	*Borzinema* De Toni 1936	
		*Coleodesmiopsis* Dutt, Datta & Gupta	
		*Coleodesmium* Borzì ex Geitler 1942	
		*Dactylothamnos* Komárek, Genuário, Fiore et Elster	
		*Dapisostemon* Hentschke, Sant'Anna et Johansen	Before Dapisostemonaceae
		*Godleya* Novis et Visnovsky	Before Godleyaceae
		*Hassallia* Berkeley ex Bornet et Flahault	
		*Kryptousia* Alvarenga, Andreote, Branco et Fiore	
		*Rexia* Casamatta, Gomez et Johansen	
		*Seguenzaea* Borzì	
		*Spirirestis* Flechtner et Johansen in Flechtner et al.	
		*Streptostemon* Sant Anna, Azevedo, Kaštovský et Komárek	
*Tolypothrix* Kützing ex Bornet et Flahault	
*Toxopsis* Lamprinou, Skaraki, Kotoulas, Economou‐Amilli et Pantazidou		Before Godleyaceae

## 3 Current State of Knowledge of the Nostocales Order in the Mexican Atlantic

The taxonomy of the group has been a source of debate throughout history; only the number of recorded species presents different data depending on the source used. According to the CYANODB2 database (Hauer and Komárek 2024), there are 1724 registered species worldwide, distributed in 451 genera, of which only 1653 are considered correct. On the other hand, in the AlgaeBase database (Guiry and Guiry 2025), there are 5625 species registered; however, this list includes fossil species, synonyms, invalid species, or species with uncertain taxonomic status; consequently, the diversity recorded today varies substantially.

Particularly in Mexico, 1123 species have been recorded (Novelo and Tavera 2019; León‐Tejera et al. 2019), of which only 164 correspond to marine environments (León‐Tejera et al. 2019), and, in turn, 148 have been recorded in the Gulf of Mexico and Mexican Caribbean (León‐Tejera, González‐Resendiz, et al. 2016; León‐Tejera et al. 2023).

**FIGURE 1:**
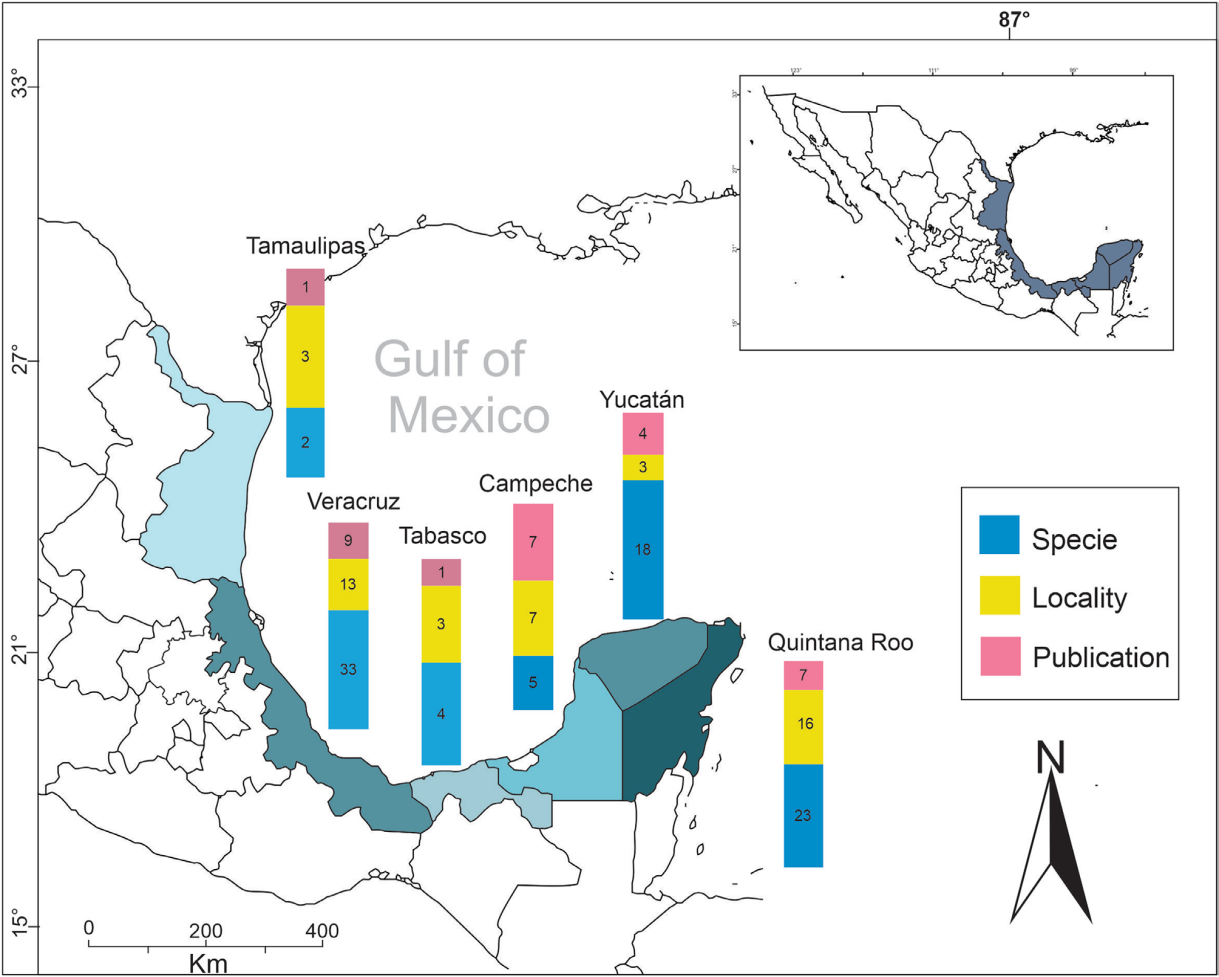
Map of the study area, Gulf of Mexico and Mexican Caribbean. Blue = number of Species; yellow = number of locations; pink = number of publications.

In the Mexican Atlantic (Figure 1), there are 22 works in which marine cyanoprokaryotes are reported (Table 2). Of the total literature collected, 18 (79%) correspond to scientific articles, four to book chapters, and one to a conference paper published in a special issue (Figure 3). Otherwise, in Ortega et al. (2001) with 23 species, León‐Tejera et al. (2009) with 14 species, Ortegón‐Aznar and León‐Tejera (2022) with 14 species, and Cabrera‐Becerril et al. (2024) with 20 species, the largest number of species known for the region is recorded. In Ortega et al. (2001), a chapter about cyanoprokaryotes shows a collection of all the records for the Gulf of Mexico and the Mexican Caribbean, including the most publications that have noted cyanoprokaryotes, especially Nostocales, from 1847 to 1996 for the study area (Table 3).

**TABLE 2:** Literature that mentions species of benthic marine cyanoprokaryotes of the Order Nostocales, for the Mexican Atlantic.

Publications	Nostocales species	Records at a generic or sp. level	Year	Type of publication	States
Günther (1990)	1	0	1990	Article	QROO
van Tussenbroek and Collado Vides (2000)	1	0	2000	Article	QROO
Ortega et al. (2001)	23	0	2001	Book chapter	All the Gulf of México and Mexican Caribbe
Mendoza‐González et al. (2017)	1	0	2007	Article	QROO
Tunnell et al. (2007)	3	0	2007	Book chapter	CAM, YUC
Ortegón‐Aznar et al. (2008)	0	1	2008	Article	YUC
León‐Tejera et al. (2009)	14	0	2009	Book chapter	All the Gulf of México
Ramírez‐Rodríguez et al. (2011)	7	0	2011	Book chapter	VER
Mateo‐Cid, Mendoza‐González, Ávila Ortiz, and Díaz Martínez (2013)	2	0	2013	Article	CAM
Mateo‐Cid, Mendoza‐González, and Fredericq (2013)	1	0	2013	Article	CAM
González‐Reséndiz et al. (2014)	0	6	2014	Article	VER
Poot‐Delgado et al. (2015)	1	1	2015	Article	CAM
Mendoza‐González et al. (2016)	6	0	2016	Article	CAM, YUC
Poot‐Delgado et al. 2017	1	2	2016	Article	CAM
Mendoza‐González et al. (2017)	4	0	2017	Article	TAB
Nava‐Olvera et al. (2017)	6	0	2017	Article	VER, QROO
González‐Resendiz, Johansen, Escobar‐Sánchez, et al. (2018)	1	0	2018	Article	VER
González‐Resendiz, Johansen, Alba‐Lois, et al. (2018)	1	0	2018	Article	VER
Johansen et al. (2021)	3	0	2021	Article	VER
Ortegón‐Aznar and León‐Tejera (2022)	14	0	2022	Article	YUC
Cabrera‐Becerril et al. (2024)	20	7	2024	Article	VER
Nava‐Olvera et al. (2024)	7	1	2024	Article	QROO
Hernández‐Casas et al. (2024)	3	0	2024	Article	QROO

Abbreviations: CAM = Campeche; QROO = Quintana Roo; TAB = Tabasco; VER = Veracruz; YUC = Yucatán.

**TABLE 3:** Literature cited in Ortega et al. (2001), from 1847 to 1996, with reported cyanoprokaryotes species.

Year	Author	Number of reported species
1847	Agardh J.	2
1847	Kützing	1
1907	Forti	2
1928	Taylor	1
1958	Huerta‐Muzquiz	2
1961	Huerta‐Muzquiz	1
1962	Humm & Hildebrand	3
1964	Kim	13
1968	Drouet	1
1972	Earle	2
1973	Drouet	2
1976	Garza‐Barrientos	4
1977	Huerta et al.	2
1978	Drouet	1
1980	Huerta & Garza‐Barrientos	6
1980	Sánchez‐Rodríguez	1
1985	Mendoza‐González & Mateo‐Cid	1
1987	Huerta et al.	4
1991	Martínez‐Lozano & Guajardo‐Ríos	3
1991	Mateo‐Cid y Mendoza‐González	5
1992	Mendoza‐González & Mateo‐Cid	3
1993	Collado‐Vides & González‐González	1
1994	Collado‐Vides et al.	2
1995	Collado‐Vides et al.	1
1995	Ortega	1
1996	Mateo‐Cid et al.	1

The chapter by León‐Tejera et al. (2009) goes back to many of the reports mentioned earlier in the chapter by Ortega et al. (2001) without adding any new findings; while it offers a more detailed review because it covers a larger area (including the Gulf of Mexico in the United States), it does not specify where the collections were made and only talks generally about where the species are found in the Gulf of Mexico.

Of all the publications (Figure 4), only those by Johansen et al. (2021) and González‐Resendiz, Johansen, Alba‐Lois, et al. (2018), González‐Resendiz, Johansen, Escobar‐Sánchez, et al. (2018) present specific information such as detailed descriptions, photomicrographs, and molecular analyses (using the 16s, 23s, and ITS markers) to describe new species, which correspond to the genera *Nunduva* González‐Resendiz, León‐Tejera & J. R. Johansen 2018, *Phylonema* Alvarenga, Rigonato, Branco, Melo & Fiore 2018 (González‐Resendiz, Johansen, Alba‐Lois, et al. 2018; González‐Resendiz, Johansen, Escobar‐Sánchez, et al. 2018), and *Kyrtuthrix* Ercegovic 1929 (Johansen et al. 2021). In the article by Cabrera‐Becerril et al. (2024), an updated list of the Nostocales cyanoprokaryotes of the State of Veracruz was presented, which includes detailed descriptions, photomicrographs of the registered species, and potential new species for the state, which require molecular analyses for their description. Of the remaining works, it is worth mentioning that Mendoza‐González et al. (2017) contribute to the first work for Tabasco and present a more exhaustive list of the cyanoprokaryotes in that state. However, the localities correspond, above all, to lagoon systems, and no other information is provided, such as morphological descriptions of the species or photomicrographs. In the articles by Nava‐Olvera et al. (2017, 2024) and those of Poot‐Delgado et al. (2015, 2017), although the group is mentioned in its relationship with other organisms and includes ecological data, they do not present descriptions or micrographs that could be useful for future comparisons. Additionally, in the works of Poot‐Delgado et al. (2015, 2017), only one species is identified at this level (Table 4); the rest of the records are maintained at the generic level (*Anabaena* sp. Bory ex Bornet & Flahault, *nom. cons.; Nostoc* sp. Vaucher ex Bornet & Flahault), so it cannot be compared with later works either.

**TABLE 4:** Taxonomic list of marine cyanoprokaryotes of the order Nostocales from the state of Veracruz and the Yucatán Peninsula.

Order	Family	Genera	Correct name	Reported as	Nomenclatural status	Reported by
Nostocales	Aphanizomenonaceae	*Trichormus*	*Trichormus propinquus* (Setchell & N.L. Gardner) Komárek and Anagnostidis (1989)	As *Anabaena propinqua* Setchell & N.L. Gardner *1919* (13)	1	15
		*Raphidiopsis*	*Raphidiopsis cuspis* (Komárek & Kling) Aguilera, Berrendero Gómez, Kastovsky, Echenique & Salerno 2018	As *Cylindrospermopsis cuspis* Komárek & Kling 1991	1	18, 19
		*Nodularia*	*Nodularia harveyana* Thuret ex Bornet & Flahault 1886		0	7, 12, 15, 17
		*Nodularia*	*Nodularia spumigena* Mertens ex Bornet & Flahault 1888		0	11, 15, 20
	Calotrichaceae	*Calothrix*	*Calothrix aeruginea* Thuret ex Bornet & Flahault		0	1,7, 15, 17
		*Calothrix*	*Calothrix confervicola* C. Agardh ex Bornet & Flahault		0	5, 7, 12, 13, 14, 15, 17
		*Calothrix*	*Calothrix contarenii* Bornet & Flahault		0	1,13, 14
		*Calothrix*	*Calothrix fonticola* Brabez 1941		0	1
		*Calothrix*	*Calothrix fuscoviolacea P. Crouan & H. Crouan ex Bornet and Flahault*		0	14
		*Calothrix*	*Calothrix longifila* Taylor (1928)		0	15
		*Calothrix*	*Calothrix parietina* Thuret ex Bornet & Flahault 1886		0	12
		*Calothrix*	*Calothrix prolifera* Flahault in Bornet & Flahault		0	1
		*Calothrix*	*Calothrix pulvinata C. Agardh ex Bornet and Flahault*		0	14

	Hapalosiphonaceae	*Fischerellopsis*	*Fischerellopsis harrisii* F.E. Fritsch 1932		0	15, 17
		*Mastigocoleus*	*Mastigocoleus testarum* Lagerheim ex Bornet & Flahault 1886		0	7, 15, 17
	Microchaetaceae	*Microchaete*	*Microchaete vitiensis* Askenasy ex Bornet & Flahault 1886		0	7, 15, 17
	Nostocaceae	*Anabaena*	*Anabaena oscillarioides* Bory ex Bornet & Flahault 1886, 233	As *Anabaena pseudoscillatoria* Bory de Saint‐Vicent (18)	1	11, 14, 15, 17, 20
		*Hydrocoryne*	*Hydrocoryne enteromorphoides* (Bornet & Flahault) Umezaki & M. Watanabe 1994	As *Hormothamnion enteromorphoides* (6, 13)	1	7, 15, 17
	Rivulariaceae	*Dichothrix*	*Dichothrix fucicola* Bornet & Flahault		0	7, 15, 17
		*Dichothrix*	*Dichothrix penicillata* Zanardini ex Bornet & Flahault 1886		0	7, 15, 17
		*Dichothrix*	*Dichothrix ramenskii* Elenkin 1922		0	13, 14
		*Dichothrix*	*Dichothrix rupicola* Collins 1901		0	7, 15, 17
		*Dichothrix*	*Dichothrix gypsophila* Bornet & Flahault 1886		0	13
		*Isactis*	*Isactis centrifuga* Bornet 1901		0	7, 15, 17
		*Isactis*	*Isactis plana* Thuret ex Bornet & Flahault 1886		0	7, 15, 17
		*Kyrthuthrix*	*Kyrtuthrix huatulcensis* León‐Tejera, González‐Resendiz, et al. (2016)		0	1
		*Kyrthuthrix*	*Kyrtuthrix munecosensis* Johansen et al. 2021		0	2
*Kyrthuthrix*		*Kyrtuthrix totonaca* Johansen et al. 2021		0	2


		*Nunduva*	*Nunduva kania* González‐Reséndiz, León‐Tejera & J.R. Johansen in González‐Reséndiz & al.		0	4
		*Nunduva*	*Nunduva komarkovae* González‐Resendiz, León‐Tejera & J.R. Johansen 2021		0	2
		*Phyllonema*	*Phyllonema ansatum* González‐Resendiz, León‐Tejera & J.R. Johansen 2018	As *Phylonema ansata* González‐Resendiz, León‐Tejera & Johansen sp. nov.(3)	2	1,3
		*Rivularia*	*Rivularia atra* Roth ex Bornet & Flahault 1886		0	11,17
		*Rivularia*	*Rivularia atra* var. *confluens* Bornet 1892		0	15
		*Rivularia*	*Rivularia bornetiana* Setchell 1895		0	13, 14
		*Rivularia*	*Rivularia litorea* G.S. An 1989		0	1
*Rivularia*		*Rivularia nitida* C.Agardh ex Bornet & Flahault 1886	0		1
*Rivularia*	*Rivularia polyotis* Roth ex Bornet & Flahault 1886	0	15	

	Scytonemataceae	*Brachytrichia*	*Brachytrichia quoyi* (C. Agardh) Bornet & Flahault	As order Oscillatoriales (18)	2	8, 15, 20
		*Scytonema*	*Scytonema crispum* C Bornet ex De Toni.		0	1
		*Scytonema*	*Scytonema hoffmannii* C.Agardh ex Bornet & Flahault 1886	*Scytonema hoffman‐bangii* in Oscillatoriales Order (18); as *Scytonema hofmannii* C. Agardh ex Bornet and Flahault (10, 15)	1, 2	6, 11, 15, 17, 20
		*Scytonema*	*Scytonema polycystum* Bornet & Flahault 1886	*As oscillatoriales Order (18)*	2	19
		*Scytonematopsis*	*Scytonematopsis crustacea* (Thuret ex Bornet & Flahault) Kováčik & Komárek 1988	As *Calothrix crustacea Thuret ex Bornet & Flahault 1886 (6, 9, 13, 18); as Scytonematopsis crustacea* (Thuret ex Bornet & Flahault) Kováčik & Komárek 1988 (12, 15, 11, 10, 8)	0	1, 5, 7, 9, 10, 11, 12, 13, 14, 15, 17, 20
		*Scytonematopsis*	*Scytonematopsis fuliginosa* (Tilden) J.J.Copeland 1936		0	1
		*Scytonematopsis*	*Scytonematopsis pilosa* (Bornet & Flahault) Umezaki & M. Watanabe 1994	As *Calothrix pilosa* Harvey ex Bornet & Flahault 1886 (6, 13)	1	1, 7, 15
Capsosiraceae	*Desmosiphon*	*Desmosiphon neocaledonicus* Bourrelly		0	1

*Note:* 1. Cabrera‐Becerril et al. (2024); 2. Johansen et al. (2021); 3. González‐Resendiz, Johansen, Escobar‐Sánchez, et al. (2018); 4. González‐Resendiz, Johansen, Alba‐Lois, et al. (2018); 5. Hernández‐Casas et al. (2024); 6. León‐Tejera et al. (2009); 7. Günther (1990); 8. Mateo‐Cid, Mendoza‐González, Ávila Ortiz, and Díaz Martínez (2013); 9. Mateo‐Cid, Mendoza‐González, and Fredericq (2013); 10. Mendoza‐González et al. (2007); 11. Mendoza‐González et al. (2016); 12. Mendoza‐González et al. (2017); 13. Nava‐Olvera et al. (2017); 14. Nava‐Olvera et al. (2024); 15. Ortega et al. (2001); 16. Ortegón‐Aznar et al. (2008); 17. Ortegón‐Aznar and León‐Tejera (2022); 18. Poot‐Delgado et al. (2017); 19. Poot‐Delgado et al. (2015); 20. Ramírez‐Rodríguez et al. (2011); 21. Tunnell et al. (2007); 22. van Tussenbroek and Collado Vides (2000). SIN column: (1) recorded as a currently synonymous name; (2) error or correction that has been made; (0) reported with the correct name.

Ortegón‐Aznar and León‐Tejera (2022), which includes an analysis of the state of knowledge of macroalgae and cyanoprokaryotes from the state of Yucatán, reported in the CONABIO database KT016 (León‐Tejera et al. 2019; León‐Tejera et al. 2023). However, the Nostocales records in the article correspond completely with the records of Ortega et al. (2001); without new records, therefore, the specific richness of cyanoprokaryotes in the state maintains the status of “prospective.”

Additionally, there are other publications that present records at a generic level for Yucatán; for example, Ortegón‐Aznar et al. (2008) register other orders and a report of *Scytonema* sp.; del Carmen Merino‐Virgilio et al. (2013) present reports for coastal areas of the state of Yucatán; however, only two correspond to marine environments: *Spirulina* sp. Agardh ex Bornet & Flahault 1892 and *Chroococcus* sp. Nägeli 1849, without further information about the morphology, locality, or details about its ecology that would allow subsequent associations with the description, as they might be new species. Herrera‐Silveira (2006), in a study about the coastal lagoons of Yucatán, mention the group in general as an important component of the phytoplankton of these environments but do not present a list of species.

In the case of Veracruz, there is the work of González‐Reséndiz et al. (2014), which deals with an analysis of the diversity of cyanoprokaryotes of Playa Muñecos but provides only generic records. In Martínez‐Arroyo et al. (2000) (not included in the book by Ortega et al. 2001), an analysis of the impact of discharges on the phytoplankton of Tuxpan, Veracruz, was presented, mentioning four cyanoprokaryotes, a non‐heterocytous filamentous species, and three unidentified “cyanophytes.*”* Additionally, in Cabrera Becerril et al. (2024), 8 species were presented at a generic level; however, they are accompanied by detailed descriptions and photographs for later comparison.

In Quintana Roo, the article by Chuc‐Contreras et al. (2012) studied the algae that grow on coral in Playa Mahahual, but it only referred to cyanoprokaryotes as “cyanophytas” without giving more details, except for their connection to different coral species. For Campeche, Soto et al. (2004) included cyanoprokaryotes in the study of phytoplankton diversity in the Campeche Bank, but only mentioned coccal and Oscillatoriales species, with some identified only by their general type. Lastly, in Tamaulipas, Poot‐Delgado and Guzmán Noz (2009) presented a congress report that talked about the salt tolerance of a coccoid cyanobacterium.

For the state of Campeche, Soto et al. (2004) mention cyanoprokaryotes as part of the phytoplankton diversity analyzed in the Campeche Bank but only mention coccal and Oscillatoriales species, some identified only at a generic level.

Finally, for Tamaulipas, there is the work of Poot‐Delgado and Guzmán Noz (2009), which itself constitutes a congress report, where the halotolerance of a coccoid cyanobacterium is mentioned.

Based on the analysis of the aforementioned works, a first updated list of the benthic marine cyanoprokaryotes of the order Nostocales, known for the Gulf of Mexico and the Mexican Caribbean, is presented, as well as their taxonomic status, nomenclatural update, and works in which they are recorded (Table 4). Forty‐five valid species belong to 19 genera and eight families, six names that have fallen into synonymy, and four corrections were recorded due to the use of invalid names or poor positioning at the taxonomic level. The most represented family was Rivulariaceae (19 species), followed by Calotrichaceae (9), Scytonemataceae (7), Aphanizomenonaceae (4), Nostocaceae (2), and Hapalosiphonaceae, Microchaetaceae, and Capsosiraceae, with one species each.

**FIGURE 2:**
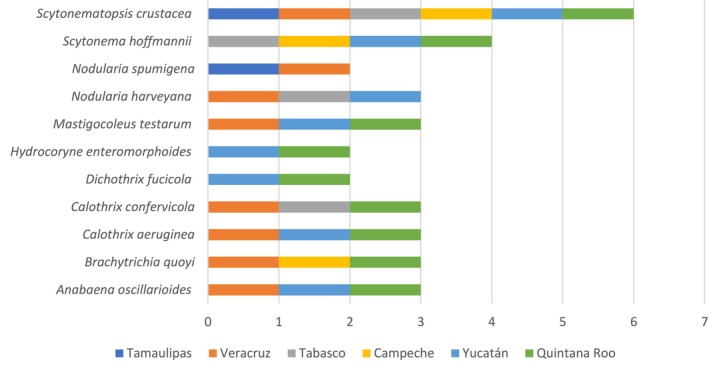
Graph Species by state. The bars represent the states in which the species are present. Only those species present in more than one state are presented. Blue = Tamaulipas; orange = Veracruz; gray = Tabasco; red = Yucatán; green = Quintana Roo.

The species with the greatest distribution within the study area were *Scytonematopsis crustacea* (Thuret ex Bornet & Flahault) Kováčik & Komárek 1988, present in all six states, followed by *Scytonema hoffmannii* C. Agardh ex Bornet & Flahault 1886, present in four states, *Anabaena oscillarioides* Bory ex Bornet & Flahault 1886, *Brachytrichia quoyi* Bornet & Flahault 1886, *Calothrix aeruginea* Thuret ex Bornet & Flahault 1886, *Calothrix confervicola* C. Agardh ex Bornet & Flahault 1886, *Mastigocoleus testarum* Lagerheim ex Bornet & Flahault 1886, and *Nodularia harveyana* Thuret ex Bornet & Flahault 1886, all present in three states (Figure 2).

**FIGURE 3:**
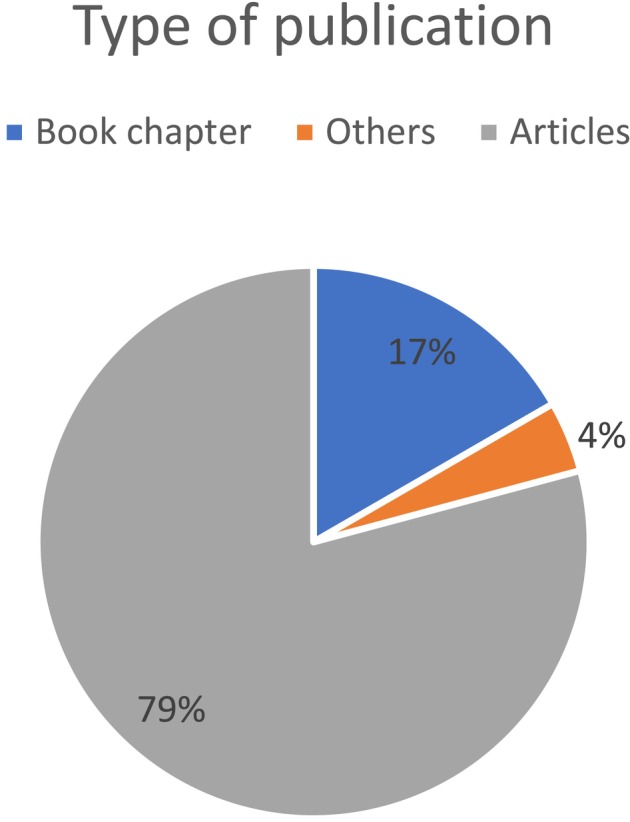
Graphic types of works that deal with cyanoprokaryotes, for the Mexican Atlantic. Gray = phycofloras 76%; Blue = Books chapter 19%; Orange = Other 5%.

**FIGURE 4:**
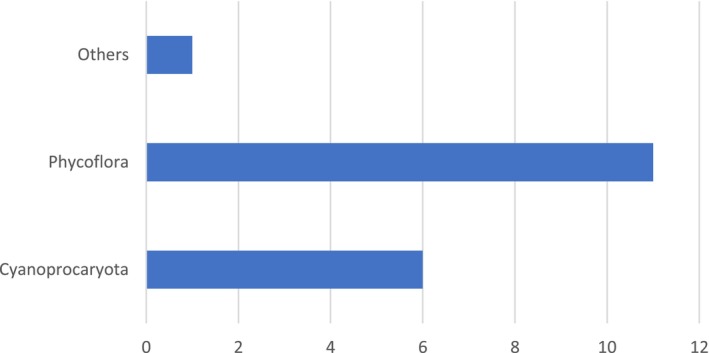
Thematic graph of the articles with reports of cyanoprokaryotas for the Mexican Atlantic. Ten phycofloras, four specific works on cyanoprokaryotas, and a report on phytoplankton.

The states that presented the highest numbers of species were (in descending order) *Veracruz (33), Yucatán (18), Quintana Roo (23), Campeche (5), Tabasco (4), and Tamaulipas (2)* (Figure 1). Likewise, the states with the highest number of associated publications were (in descending order): Veracruz (9), Campeche (7), Yucatán (5), Quintana Roo (7), Tabasco (1), and Tamaulipas (1) (Figure 1).

## 4 Taxonomic Problems at the Family, Genus, and Species Level in the Order Nostocales

The information provided below includes very detailed insights into the taxonomic challenges faced by certain taxa (which at the time presented taxonomic problems of different kinds), ranging from the family level to the species level. The descriptions pertain to those recorded in the Mexican Atlantic and Caribbean.

### 4.1 Family Scytonemataceae

The family Scytonemataceae, which includes *Petalonema* Berkeley ex Correns 1887, *Sctynonematopsis* Kiseleva 1930, *Sctyonema*, and *Brasilonema* Fiore, Sant'Anna, Azevedo, Komárek, Kaštovský, Sulek, and Lorenzi 2007, is defined from molecular evidence. Komárek (2013) mentions that there are still species contained in the four current genera, which most likely must be transferred to new genera segregated from the preexisting ones or described as new species. Likewise, Komárek (2013) proposed that other genera, such as *Scytonema* sect. *Myochrotes* Bornet et Flahault (1887) and Chakia J. Komárková, E. Zapomelová, and J. Komárek (2013), probably belong to Symphyonemataceae.

On the other hand, *Brasilonema*, although with a molecularly defined phylogenetic identity (Vaccarino and Johansen 2012; Komárek 2013), was mentioned as the genus of “tropical *Scytonema*” and included a single unidentified species for Europe and 11 species of *Scytonema* outside of Europe, including a Mexican freshwater species (*B. tolantogense* Becerra‐Absalón & Montejano (Becerra‐Absalón et al. 2013)) that does not present sufficient information on other species of the genus.

*Scytonema sect. Myochrotes*, traditionally contained within and outside of *Scytonema* on several occasions (it has been considered a subgenus, although its morphology is very similar to *Scytonema*), has also been described as similar to *Petalonema*; the three genera require revision, according to the author, due to these morphological similarities and the lack of molecular analyses that clarify their positions (Komárek 2013). *Scytonematopsis*, within this family, represented at that time another case in which only the sequences of *S. contorta* M. A. Vaccarino & J. R. Johansen 2011 with more than 15 species described at the time of publication; only two species are fully accepted, 11 more in the section outside Europe with minimal information both morphological and ecological, and six species as uncertain, showing again the problem faced by the identification of species in tropical environments and especially in México.

#### 4.1.1 *Arthrosiphon* Kützing 1843, *nom. inval*

It is considered an invalid name due to the date of its description, prior to Bornet and Flahault (1886–1888), composed of three species. *A. alatus* Rabenhorst (1865), *nom. inval*., is currently heterotypic synonymous with *Petalonema alatum* (Borzì ex Bornet & Flahault). Wolle 1887, *A. densus* A. Braun 1849 is currently heterotypic synonymous with *Petalonema densum* (Bornet ex Bornet & Flahault) Migula 1907 and *A. grevillei* Kützing ex Frank 1886, which is described as correct status since it is part of *Arthrosiphon* Kützing ex Frank 1889, considered within the starting point (Guiry and Guiry 2025). However, in Komárek (2013), the genus is considered heterotypic synonymous with *Petalonema* Berkeley ex Correns. In Hauer and Komárek (2024), it is recognized as a heterotypic synonym of *Petalonema*.

#### 4.1.2 *Drilosiphon* Kützing 1843, *nom. inval*.

The AlgaeBase database (Guiry and Guiry 2025) describes two species, but the genus remains as *nom. illeg*., since its description dates back before the starting point, and it is mentioned that more studies are required to describe its status. *D. julianus* Kützing 1847, *nom. inval*., is a heterotypic synonym of *Scytonema julianum* Meneghini ex B.A. Whitton 2011, and *Drilosiphon muscicola* Kützing 1843 remains invalid. In Hauer and Komárek (2024), it also remains with invalid status for the same reason mentioned above: it is recognized as a heterotypic synonym of *Scytonema*.

#### 4.1.3 *Scytonema hoffmannii* C. Agardh ex Bornet & Flahault 1886

The name has been a source of controversy as it has been cited in different ways and has several heterotypic synonyms today. (*S. hofman‐bangii* C. Agardh 1812; *Symphyosiphon hofmannii* Kützing 1849; *S. hansgirgianum* P. Richter 1884; *S. hoffmannii var. hansgirgianum* Hansgirg 1892; *S. hoffmannii* f. *hansgirgianum* (Hansgirg) Kossinskaja 1938). Over the course of the 20th century, revisions have accepted some species and discarded others. In the work of Ortega et al. (2001), it is mentioned that name variants had been reported: *S. hofmanni* (Forti 1907), *S. hofmannii* (Drouet 1973; Huerta‐Múzquiz et al. 1987), and *S. hoffmanii* (Mateo‐Cid and Mendoza‐González 1991; Mendoza‐González and Mateo‐Cid 1992; Collado‐Vides et al. 1994). In the same work by Ortega et al. (2001), the authors indicated that Silva et al. (1996) suggested that the names of “*S. hofmanni”* and “*S. hoffman‐bangii”* were not correct, and due to the starting point of cyanoprokaryotes in Bornet & Flahault (1886–1888), it was suggested that only “*S. hofman‐bangii” was correct*.

Regarding the name, Guiry and Guiry (2025) described its appearance for the first time in the works of Agardh in 1812, in which the specific epithet of “*hofman–bangii” was used*; later, in 1817, Agardh himself (and later authors) described the genus as “*hofmanni*.”

Since the “starting point” has been established in Bornet & Flahault (1886–1888), where it was determined as *S. hoffmannii* (unless the starting point is moved), then the name would return to *S. hofman‐bangii*, C. Agardh, as it would be the first record.

The names *Brachytrichia quoyi* Bornet & Flahault, *S. hoffman‐bangii*, and *S. polycystum* were reported by Nava‐Olvera et al. (2017) as part of Oscillatoriales, although according to the classification systems of Hauer and Komárek (2024) and Guiry and Guiry (2025), they belong to the order Nostocales. In Table 2, their position is corrected.

### 4.2 Family Rivulariaceae y Calotrichaceae

*The Rivulariaceae* family, as described by Komárek in 2013, comes from splitting off from the Nostocaceae family because of the physical and genetic traits of *Rivularia* and *Calothrix*. From this, *Gloeotrichia* J. Agardh ex Bornet & Flahault 1886 is mentioned as the genus that seemed to be phylogenetically related to the Nostocaceae family. However, this hypothesis was only supported by the sequence of a single species ( *G. echinulata* P. G. Richter 1894), so reference was made to the need to carry out more in‐depth analyses to define its position. On the other hand, *Calothrix*, which is one of the largest groups in this family with 30 species in Europe and 60 elsewhere until 2013, has caused confusion because it is made up of different types, showing that the traditional *Calothrix* group was split from those that had a hair on the end.

Although some attempts were made to reclassify the family (Castenholz et al. 2001), separating marine *Calothrix* as *Rivularia* and freshwater *Calothrix* as *Calothrix*, this separation had no long‐term effect (Komárek 2013).

In the system of Strunecký et al. (2023), the family has disintegrated by placing *Calothrix* back to Rivulariaceae. Saraf et al. (2019) proposes creating a new family called Calotrichaceae, as mentioned in the Hauer and Komárek (2024) system, because genetic information backs up this separation. However, Strunecký et al. (2023) suggested not considering it as a family distinct from Rivulariaceae if there is no in‐depth analysis of the “typical” species of *Calothrix*. Kumar et al. (2023) mention that Calotrichaceae is separated from Rivulariaceae, being sister clades, and present two new genera, *Fulbrightiella* N. Kumar, P. Singh & J.R. Johnson 2022 and *Sherwoodiella* J.R. Johansen & P. Singh 2022, considered within the classification of Hauer and Komárek (2024), again supporting the separation of the two families; however, these genera are not considered within the classification system of Strunecký et al. (2023).

#### 4.2.1 *Calothrix cyanea* (J. Agardh) 1847

Agardh (1847, 1848) reported this species for the first time, and it was recovered in the work of Ortega et al. (2001). However, according to Pedroche (2022), the name is prior to the starting point considered in Bornet & Flahault (1886–1888), as part of the Nostocaceae family in that classification system. Nevertheless, these authors have placed it as a species with uncertain status. Additionally, it has not been considered again in subsequent works, nor in other classification systems such as Komárek (2013), Guiry and Guiry (2025) or Hauer and Komárek (2024).

#### 4.2.2 *Calothrix juliana* Bornet & Flahault ex Gomont 1892

It was recorded in the work of Ortega et al. (2001) for Arrecife Alacranes in the state of Yucatán; the original report comes from the doctoral thesis of Kim (1964), one of the sources considered in Ortega et al. (2001). The taxon in question has been moved from Nostocales and is currently found as *Homoeothrix juliana* (Bornet & Flahault ex Gomont) Kirchner 1898, of the family Microcoleaceae, in order Oscillatoriales. The combination of *H. juliana* (Bornet & Flahault ex Gomont) Kirchner 1898 and *C. juliana* as its basonym was proposed in the work of Vodenicharov (1971), after the arrangement described in Geitler's work in 1930 (Gärtner 2018).

#### 4.2.3 Dasyactis Kützing 1843

The genus *Dasyactis* contains 13 species, of which only two are considered recognized synonyms: D. *kunzeana* Kützing, 1843 (heterotypic synonymous of *Rivularia kunzeana* Forti) and *D. plana* (Harvey) Kützing, *nom. inval*. (heterotypic synonymous of *Isactis plana* Thuret ex Bornet & Flahault). According to Guiry and Guiry (2025), the rest of the species remain of uncertain status due to publication dates prior to the starting point in 2014. In Hauer and Komárek (2024), it is recognized as heterotypic synonymous of *Calothrix*.

#### 4.2.4 *Diplotrichia* J. Agardh 1843

It was described with a single species *D. polyotis* J. Agardh 1842, which is currently considered heterotypic synonymous with *Rivularia polyotis* Roth ex Bornet & Flahaul 1886t (Guiry and Guiry 2025). In Komárek (2013) the existence of this genus is not recognized, within *Rivularia polyotis*, no synonyms are described. In Komárek (2013) the existence of this genus is not recognized, within *Rivularia polyotis*, no synonyms are described. In the classification system of Hauer and Komárek (2024), it is recognized as a synonym of *Rivularia*, as it was described prior to the starting point.

#### 4.2.5 *Geocyclus* Kützing 1843

Described with a single species in Rabenhorst (1865), the genus is considered invalid, due to its origin before the starting point. It is sometimes recognized as *Geacyclus* due to the way its name is printed in different issues; in the system of Guiry and Guiry (2025), it is not recognized. In Hauer and Komárek (2024), it is recognized as heterotypic synonym of *Rivularia*.

#### 4.2.6 *Inomeria* Kützing 1845 *nom inval*.

Rabenhorst (1865) described four species, of which only *I. umbilicata* Nägeli 1849 is recognized as a heterotypic synonym with *Phormidium* (Guiry and Guiry 2025), while *I. brebissoniana* Kützing and *I. roemeriana* Kützing 1845 remain with uncertain status in this classification system. However, in Komárek (2013) the genera status remains invalid. In Hauer and Komárek (2024), it is recognized as a synonym of *Schizothrix* within the Order Oscillatoriales.

#### 4.2.7 *Kyrtuthrix* Ercegovic, 1929

This case is of special attention, as this genus was considered part of the Scytonemataceae family; however, it is currently found in the Rivulariaceae family (Strunecký et al. 2023; Hauer and Komárek 2024). Komárek (2013) described the generic problem, classified as Scytonemataceae due to its “branching” pattern (although these are folds of the trichomes, that is, the trichome bends on its same axis, generating a fold pattern with the shape of “u”, non‐branching type Scytonematopsis, where the trichomes meet each other in their direction of growth, and changing direction, forming a false branching) and its trichomes are isopolar with intercalary heterocyte*s*. However, only two species had been described up to that point, and without molecular sequences, so the morphological similarity of both was recorded as a possible case of synonyms (*K. dalmática* Ercegovic and *K. maculans* (Gomont) I. Umezaki).

This case is relevant for Mexican diversity because of the species that have been recorded, three for benthic marine environments: *K. totonaca* González‐Resendiz, León‐Tejera & J.R. Johansen, *K. munecosensis* González‐Resendiz, León‐Tejera & J.R. Johansen for Veracruz state, and *K. huatulcensis* León‐Tejera, González‐Resendiz, and Johansen identified *K. huatulcensis* for the state of Oaxaca (León‐Tejera, González–Resendiz, et al. 2016; González‐Resendiz, Johansen, Alba‐Lois, et al. 2018; González‐Resendiz, Johansen, Escobar‐Sánchez, et al. 2018). This genus is under‐researched not only in Mexico but also worldwide, exhibiting distinctive morphology characterized by the formation of “loops” or “u”‐shaped turns of the trichomes, with apices and cellular shapes that vary.

#### 4.2.8 *Limnactis* Küzing ex Bornet & Flahault 1843

This genus is currently considered synonymous with *Rivularia* (Guiry and Guiry 2025), and is composed of five species: *L. dura* Kützing ex Bornet & Flahault (heterotypic synonyms of *Rivularia dura* Roth ex Bornet & Flahault), *L. flagellifera* Kützing 1852 (heterotypic synonyms) *Rivularia minutula var. flagellifera* (Kützing) Hansgirg; and *L. lyngbyana* Kützing 1823, *L. minutula* Kützing 1843, and *L. rivularis* Kützing with uncertain status for which further research is suggested to determine their status, all three lack a type specimen (Guiry and Guiry 2025). In Komárek (2013), it is not recognized as synonymous with *Rivularia* species reported in Algaebase. In Hauer and Komárek (2024), the name is corrected to *Limnanthe* Kützing; it is recognized as heterotypic synonymous with *Aphanizomenon* because it was described before the work of Bornet and Flahault (1886/1888).

#### 4.2.9 *Phylonema ansata* a *Phyllonema ansatum* González‐Resendiz, León‐Tejera & J.R. Johansen 2018

In this case, said species has been corrected in the AlgaeBase database (Guiry and Guiry 2025) as *Phyllonema ansatum*, while in the CyanoDB2 database (Hauer and Komárek 2024), it continues as *P. ansata* , as reported by Alvarenga et al. (2016) for the genus *Phyllonema* (since the name comes from phyllon which means leaf), with one species: *P*. *aviceniicola* Alvarenga, Rigonato, Branco, Melo & M.F. Fiore. In both cases, *P. ansata* or *P. ansatum* appear as correct and accepted names.

### 4.3 Family Nostocaceae

In the classification systems prior to Komárek (2013), the Nostocaceae family was also already represented by several clades, and although they were not well defined at that time, some of the genera could be distinguished by a morphology very different from that of the type, *Nostoc*, which would eventually be reflected in other subsequent studies (Komárek 2013; Strunecký et al. 2023; Kabirnataj et al. 2020; Bagchi et al. 2017).

In Komárek et al. (2014), the authors once again took up the advances of that time, proposing a new revised classification system in which the order Nostocales is composed of 12 families and 118 genera, adding Gloeotrichiaceae based on molecular analyses of the 16S gene. Before this, the latter's members were in Hapalosiphonaceae, but in this system, they were found to be phylogenetically related to Calothrix and other Nostocaceae members. In this study, Nostocaceae was identified as polyphyletic; the genus Nostoc has been subdivided into several genera, including *Mojavia* Baldarelli, Pietrasiak & J.R. Johansen and *Demonostoc* Pecundo & Tao Chen.

#### 4.3.1 *Sphaerozyga* C. Agardh ex A.B.Frank, 1886

It is a genus recognized by Guiry and Guiry (2025), conformed of 26 species; however, except for *S. fallax* Ripart, there are no subsequent records of all species. The last update in AlgaeBase, in 2015 (Guiry and Guiry 2025), reveals that 13 species are currently synonymous with other species, and the remaining 12 are of uncertain taxonomic status. The author Komárek (2013) only recognizes one species, *S. saccata* Wolle, as a heterotypic synonym of *Wollea saccata* Bornet & Flahault. Komárek (2013) only recognizes one species *S. saccata* Wolle, as a synonym of *W. saccata* Bornet & Flahault. In the CYANODB database of Hauer and Komárek (2024) it is recognized as a heterotypic synonym of *Anabaena*, since its status is invalid due to having been described before the starting point of the Nostocales.

#### 4.3.2 *Hormosiphon* Kützing 1843, *nom. inval*.

In AlgaeBase (Guiry and Guiry 2025), this generic name is considered invalid or illegitimate, composed of five species, of which four are synonyms (*Hormosiphon antillarum* P. Crouan & H. Crouan homotypic synonymous of *Nostoc antillarum* P. Crouan & H. Crouan) P. Crouan & H. Crouan, *H. granularis* Kützing homotypic synonymous of *Nostoc granulare* (Kützing) Rabenhorst, *H. margaritaceus* Kützing homotypic synonymous of *N. margaritaceum* (Kützing) Rabenhorst, *H. tenuissimus* Kützing homotypic synonymous of *N. tenuissimum* (Kützing) Rabenhorst and one is of uncertain status, as is the case of *H. furfuraceus* Kützing, *nom. inval*., now considered an invalid name, due to the lack of a type designating the gender. In Hauer and Komárek (2024), it is recognized as a synonym for *Nostoc*, because it was described prior to the starting point.

#### 4.3.3 *Nostoc caladarium* H.C. Wood, *nom. inval*. 1868

Registered by Ramírez‐Rodríguez et al. (2011) for the state of Veracruz. This species was reported for the first time in Wood (1868); however, as in the previous case, being prior to the starting point of the Nostocales, which is considered in Bornet and Flahault (1886/1888), the name loses validity in the classification system.

According to Algae Base (Guiry and Guiry 2025), in Bornet and Flahault (1886/1888), the species is found in the “*Species Inquerendae*” section (species to explore or investigate) so a more in‐depth review has been pending.

#### 4.3.4 *Spermosira* Kützing ex Frank 1886

Now separated into *Nodularia* and *Trichormus* (Ralfs ex Bornet & Flahault) Komárek & Anagnostidis, it was described in Kützing (1847) with reference to the name *S. vriescana* Kützing 1847. This genus was described prior to the starting point, where it was described as a separate taxon, due to the presence of akinetes that formed in groups, unlike *Nodularia*. However, Bornet and Flahault (1888–1889) considered that this characteristic was not enough to separate both genera, which is why it was merged with *Nodularia*.

In Guiry and Guiry (2025), it is described as a genus, considered invalid, and it is mentioned that further analysis is required to resolve its taxonomic position. Five species of the genus are recorded in this database: *Spermosira hallensis* Janczewski (currently heterotypic synonymous of *Trichormus hallensis* (Bornet & Flahault) Komárek & Anagnostidis) *S*. *harveyana* Thwaites, *S. litorea* Kützing, and *S. major* . Currently, in Hauer and Komárek (2024), *S*. *macrospora* Reinsch, *nom. inval* (because it is prior to the starting point), is described as a species with taxonomic problems, recognized as a synonym of *Nodularia*. It should be noted that of the three species initially described in Kützing (1847), *S. vriescana* is not subsequently recognized by any of the classification systems.

### 4.4 Family Hapalosiphonaceae

Hapalosiohonaceae, which is composed of *Fischerella* (Bornet & Flahault) Gomont, *Westiellopsis* Janet, *Nostochopsis* H.C. Wood ex Bornet & Flahault, *Hapalosiphon* Nägeli ex Bornet & Flahault, *Mastigocladus* Dickie ex Forti, and *Mastigocoleus* Lagerheim ex Bornet & Flahault in Komárek (2013), were each forming a different family, because they form a single clade related to Nostocaceae.

#### 4.4.1 *Fischera* Schwabe, 1836, *nom. Illeg*

It is considered illegitimate due to its origin prior to the starting point and described with a species *F. thermalis* Schwabe nom. invalid. In the Guiry and Guiry (2025) classification system, two species are recognized: *F. thermalis* heterotypic synonym of *Fischerella thermalis* Gomont and *F. muscicola* Thuret, *nom. inval*., currently heterotypic synonym of *Fischerella muscicola* Gomont, genus recognized as legitimate. In Hauer and Komárek (2024), it is recognized as heterotypic synonym of *Fischerella* (Bornet & Flahault) Gomont.

### 4.5 Family Tolypothrichacea

The Tolypothrichaceae family, composed of *Coleodesmium* A. Borzì ex L. Geitler, *Hassalia* Trevisan, *Tolypothrix* Kützing ex Bornet & Flahault, *Rexia* Casamatta, Gomez & J.R. Johansen, *Spirirestis* V.R. Flechtner & J.R. Johansen, and *Dactylothamnos* Fiore & Genuario, excludes *Seguenzaea* Borzì and *Streptostemon* Sant'Anna, Azevedo, Kaštovský & Komárek, which, despite having a morphology very similar to the other genera, have phylogenetic relationships that are little known but are closer to Scytonemataceae.

### 4.6 Family Symphyonemataceae

#### 4.6.1 *Lophopodium* Kützing ex Rabenhorst, 1865 *nom. inval*.

Described in Rabenhorst (1865) with four species: *Lophopodium incrustatum* Rabenhorst, *L. crustaceaum* Rabenhorst, *L. barbatum* , and *L. hospitum* (Kützing) Rabenhorst. In the classification system of Guiry and Guiry (2025), two species were recognized, *L. hospitum* and *L. sandvicense* Nordstedt, both with synonymous status. *L. hospitum* was registered as homotypic synonymous with *Dasyactis hospita* (Kützing) P. Crouan & H. Crouan, which is no longer recognized as valid in the system; and *L. sandvicense* is registered as heterotypic synonymous with *Calothrix sandvicensis* Schmidle.

#### 4.6.2 *Meryzomiria* Kützing 1843

It is recognized in Algaebase with a single species, *Merizomyria laminosa* Kützing 1843, with correct status (Guiry and Guiry 2025), grouped in its classification system to a set of species designated as *incertae sedis*, from subclass to family. Currently, the genus remains unaccepted in other classification systems; according to Guiry and Guiry (2025), there is a report from 2022 of the species in an article by Nguyen et al. (2022), on *Meristotheca lysonensis* Nguyen et al. (2022) from Vietnam.

#### 4.6.3 *Mastigothrix* Kützing 1843

It was described in Rabenhorst (1865) with two species, *M. fusca* Kützing 1843 *nom. inval*. and *M. aeruginea* Kützing 1843. It is taken up in Guiry and Guiry (2025); however, it is rewritten as *Mastichothrix* Kützing (1847). In the classification system, three species are recognized, in addition to those previously referred to as *M. longissima* P.P. Crouan & H. Crouan; only *M. fusca* is recognized as heterotypic synonymous with *Calothrix fusca* Bornet & Flahault; the other two species are considered taxonomic problems. In this same classification system, 2 species of the genus are described, *Mastigothrix turgida* Wolle 1877 and *Mastigothrix fibrosa* Wood, nom. invalid 1869, both of which require investigation since they are not considered valid. The type of the species is described in Silva‐Pineda (1977) for the state of Chiapas, Mexico, in a work on fossil Chlorophytes (Guiry and Guiry 2025).

## 5 Discussion

The taxonomy of the entire group has gone through many changes throughout 300 years of history, thanks, in part, to the gradual development of more and better tools that allowed us to observe the species more closely. The order Nostocales has been around since people started studying nature, and it is especially recognized for having important structures called heterocytes and akinetes.

One of the main problems associated with the taxonomy of cyanoprokaryotes is that there is no other identification basis for the species in these environments, which is why, commonly, in the process of contrasting morphological characters, numerous inconsistencies and non‐correspondences were generated, such as is the case for Mexican works, especially in marine, brackish, or extreme environments.

The knowledge of the diversity and special richness of cyanoprokaryotes in Mexico, particularly those from marine, brackish, or extreme environments (such as hypersaline ones), is notably underestimated. Although there are some works that deal with the group or some works in which an effort has been made to compile all the reports for the Mexican coasts, there are very few, and even fewer for the specific case of the Nostocales Order (Ortega et al. 2001; León‐Tejera et al. 2009).

Of the 18 articles reviewed (Table 2), 11 correspond to studies of macroalgae; however, cyanoprokaryotes are included indirectly; three deal with the group specifically, and the remaining one constitutes a work on phytoplankton ecology, which, although it mentions cyanoprokaryotes, does not provide additional information about them. It is worth mentioning that, in the case of Yucatán, although many species have been recorded, the specific richness is considerably underestimated, since these records correspond to only three localities in the entire state, and the majority correspond to Kim's Ph.D. thesis (Kim 1964) from Arrecife Alacranes; likewise, in Campeche, the records correspond to five locations.

Of the 18 reviewed articles, seven correspond to specific publications on cyanoprokaryotes (Figure 4), such as those by Johansen et al. (2021), González‐Resendiz, Johansen, Alba‐Lois, et al. (2018); González‐Resendiz, Johansen, Escobar‐Sánchez, et al. (2018), Cabrera‐Becerril et al. (2024), and Nava‐Olvera et al. (2024). These works, which represent 33% of the publications, and considering that the Gulf Coast and the Yucatán Peninsula correspond to 3294 km, show few studies on richness or any topic directly linked to cyanoprokaryotes. The works of Johansen et al. (2021) and González‐Resendiz, Johansen, Alba‐Lois, et al. (2018); González‐Resendiz, Johansen, Escobar‐Sánchez, et al. (2018) deal with new species from three genera (*Phylonema, Nunduva*, and *Kyrtuthrix*), using molecular tools. In the case of Cabrera‐Becerril et al. (2024), 20 species are recorded, of which at least 17 are new records for Mexico and the state; however, these four works correspond to the state of Veracruz. For the state of Quintana Roo, the study by Nava‐Olvera et al. (2024) documents 7 species without providing microphotographs or descriptions of the recorded species but emphasizes aspects of the group's ecology. The above indicates that there is considerable potential for describing new species richness and even new species along the coastline, given that approximately 33 species for the state of Veracruz derive from four articles, while seven for the state of Quintana Roo derive from a single article. One can also mention the case of the state of Yucatán, where species richness is concentrated in the Parque Nacional Arrecife Alacranes, while the coastal shoreline is completely unexplored. More specialized studies are needed, incorporating a multifaceted approach that covers increasingly more regions in the different states, to obtain a comprehensive perspective of specific richness.

The genera *Anabaena*, *Nostoc*, *Rivularia*, and *Scytonema* were among those that have been described from the beginning in the different classification systems; therefore, they are also the genera that have undergone major taxonomic changes and still contain problematic species in the current affairs that require in‐depth revisions. With the introduction of molecularly assisted alpha taxonomy (Cianciola et al. 2010) to diversity studies, classification systems are increasingly stable, robustly reflecting the evolutionary history of species and their phylogenetic relationships. However, there is still a lot of basic science work to be done on the Mexican coast, since the group remains unexplored. So, to ensure the group's specific richness, it is vital to document and analyze Mexican populations and their morphology.

On one hand, there are no detailed descriptions of the physical features and cell sizes that match the Mexican phycoflora, and the only identification sources are European studies that do not completely align with the native Mexican plants. On the other hand, the implementation of the molecular approach as a tool for the delimitation of species and the establishment of phylogenetic relationships between them has been limited (León‐Tejera, González–Resendiz, et al. 2016; González‐Resendiz et al. 2013; González‐Resendiz et al. 2019; González‐Resendiz, Johansen, Alba‐Lois, et al. 2018; González‐Resendiz, Johansen, Escobar‐Sánchez, et al. 2018; Johansen et al. 2021), so there are various taxonomic problems within the group, which are reflected in an unstable and not very robust classification system.

With the introduction of molecularly assisted alpha taxonomy (i.e., the field of systematics responsible for naming and describing new species) (Cianciola et al. 2010) to diversity studies, classification systems are increasingly stable, robustly reflecting the evolutionary history of species and their phylogenetic relationships (Johansen et al. 2021). However, there is still a lot of basic science work to be done on the Mexican coast, since the group remains unexplored. Therefore, it is important to establish the record and analysis of Mexican populations, as well as the knowledge of their morphology, to be able to provide certainty of the specific richness of the group. This requirement is evident in the publications of Mendoza‐González et al. (2017) and Cabrera‐Becerril et al. (2024). The first effort of this kind for Tabasco state documents 26 previously unrecorded cyanoprokaryotes, while the second enhances the specific richness of Veracruz by 57% (one of the most visited states by universities).

However, as classical literature is about European work, the diversity described corresponds to temperate environments, leaving out species from tropical or subtropical environments. One of the main associated problems is that there is no other identification basis for the species in these environments, so, commonly, in the process of contrasting morphological characters, numerous inconsistencies and non‐correspondences are generated, as is the case for the Mexican works, especially from marine, brackish, or extreme environments. Likewise, it is estimated that the known diversity will increase with the detailed study of the native flora through the description of new species and the taxonomic adjustments that it entails, allowing, in the future, robust basic knowledge that facilitates the beginning of work of another nature, whether ecological or even biotechnological.

## Author Contributions

**Ernesto Cabrera‐Becerril:** conceptualization (equal), investigation (equal), resources (equal), writing – original draft (equal), writing – review and editing (equal). **Annie May Ek García‐García:** conceptualization (equal), investigation (equal), methodology (equal), writing – original draft (equal), writing – review and editing (equal). **María Luisa Núñez Resendiz:** supervision (equal), writing – original draft (equal), writing – review and editing (equal). **Abel Sentíes:** project administration (equal), writing – review and editing (equal). **Kurt M. Dreckmann:** writing – review and editing (equal).

## Conflicts of Interest

The authors declare no conflicts of interest.

## Supporting information


Appendix S1.

## Data Availability

The data that support the findings of this study are openly available.

## References

[ece371826-bib-0001] Agardh, J. G. 1847. “Nya Alger Från Mexico. Öfversigt af Kongl.” Vetenskaps‐Adademiens Förhandlingar, Stockholm 4: 5–17.

[ece371826-bib-0002] Agardh, J. G. 1848. “ *Anadema*, Ett Nytt Slägte Bland Algerne. Öfversigt af Kongl.” Vetenskaps‐Akademiens Förhandlingar, Stockholm 1846: 1–16.

[ece371826-bib-0003] Alvarenga, D. O. , J. Rigonato , L. H. Z. Branco , I. S. Melo , and M. F. Fiore . 2016. “ *Phyllonema aviceniicola* gen. nov., sp nov and *Foliisarcina bertiogensis* gen. nov., sp nov., Epiphyllic Cyanoprokaryote Associated With *Avicennia schaueriana* Leaves.” International Journal of Systematic and Evolutionary Microbiology 66: 689–700. 10.1099/ijsem.0.000774. 26582479

[ece371826-bib-0004] Anagnostidis, K. , and J. Komárek . 1985. “Modern Approach to the Classification System of *Cyanophytes* 1—Introduction.” Archhiv für Hydrobiologie/Algological Studies 38–39: 291–302.

[ece371826-bib-0005] Anagnostidis, K. , and J. Komárek . 1988. “Modern Approach to the Classification System of Cyanophytes 3—*Oscillatoriales* .” Archhiv für Hydrobiologie/Algological Studies 50–53: 327–472.

[ece371826-bib-0006] Anagnostidis, K. , and J. Komárek . 1990. “Modern Approach to the Classification System of Cyanophytes 5—*Stigonematales* .” Archhiv für Hydrobiologie/Algological Studies 59: 1–73.

[ece371826-bib-0007] Bagchi, S. N. , N. Dubey , and P. Singh . 2017. “Phylogenetically Distant Clade of *Nostoc*‐Like Taxa With the Description of *Aliinostoc* gen. nov. and *Aliinostoc morphoplasticum* sp. nov.” International Journal of Systematic and Evolutionary Microbiology 67, no. 9: 3329–3338. 10.1099/ijsem.0.002112. 28868999

[ece371826-bib-0008] Becerra‐Absalón, I. , B. Rodarte , K. Osorio , L. Alba‐Lois , C. Segal‐Kischinevzky , and G. Montejano . 2013. “A New Species of *Brasilonema* (Scytonemataceae, Cyanoprokaryota) From Tolantongo, Hidalgo, Central Mexico.” Fottea, Olomouc 13, no. 1: 25–38. 10.5507/fot.2013.003.

[ece371826-bib-0009] Bold, H. C. , and M. J. Wynne . 1978. Introduction to the Algae: Structure and Reproduction. 706 pp. Prentice‐Hall.

[ece371826-bib-0011] Bornet, É. , and C. Flahault . 1886/1888. “Revision des Nostocacées Hétérocystées Contenues dans les Principaux Herbiers de France (Quatrième et Dernier Fragment).” Annales des Sciences Naturelles, Botanique, Septième Série 7: 177–262.

[ece371826-bib-0130] Borzi, A. 1914. “Studi Sulle Mixoficee.” Nuovo Giornale Botanico Italiano Serie 21: 320–360.

[ece371826-bib-0012] Cabrera‐Becerril, E. , A. M. E. García‐García , M. L. Núñez‐Resendiz , K. M. Dreckmann , and A. Sentíes . 2024. “Diversity of Marine Benthic Species of Nostocales (Cyanoprokaryote) in Veracruz, Mexico.” Botanical Sciences 102, no. 2: 561–585. 10.17129/botsci.3391.

[ece371826-bib-0013] Castenholz, R. W. 2015. “Oxygenic Photosynthetic.” In Bacteria. Bergey's Manual of Systematics of Archaea and Bacteria, edited by W. B. Whitman , 1–6. John Wiley & Sons, Inc. 10.1002/9781118960608.cbm00020.

[ece371826-bib-0014] Castenholz, R. W. , A. Wilmotte , M. Herdman , et al. 2001. “Phylum BX. Cyanoprokaryote.” In Bergey's Manual of Systematic Bacteriology, edited by D. R. Boone , R. W. Castenholz , and G. M. Garrity , 473–599. Springer. 10.1007/978-0-387-21609-6_27.

[ece371826-bib-0015] Cavalier‐Smith, T. 2002. “The Neomuran Origin of Archaebacteria, the Negibacterial Root of the Universal Tree and Bacterial Megaclassification.” International Journal of Systematic and Evolutionary Microbiology 52, no. 1: 7–76. 10.1099/00207713-52-1-7. 11837318

[ece371826-bib-0016] Chuc‐Contreras, A. , I. Ortegón‐Aznar , A. Tuyub Mota , and J. Suárez Salazar . 2012. “Cambio de Fase Coral‐Algas en el Arrecife de Coral de Mahahual, en el Caribe Mexicano.” In Proceedings of the 64th Gulf and Caribbean Fisheries Institute, vol. 64, 28–31. 10.13140/2.1.3533.0562.

[ece371826-bib-0017] Cianciola, E. N. , T. R. Popolizio , C. W. Schneider , and E. Lane . 2010. “Using Molecular‐Assisted Alpha Taxonomy to Better Understand Red Algal Biodiversity in Bermuda.” Diversity 2: 946–958. 10.3390/d2060946.

[ece371826-bib-0018] Collado‐Vides, L. , and J. González‐González . 1993. “Macroalgas del sistema lagunar de Nichupté, Quintana Roo.” In Biodiversidad Marina y Costera de México, edited by S. I. Salazar‐Vallejo and N. E. González , 752–760. CONABIO/CIQRO.

[ece371826-bib-0019] Collado‐Vides, L. , J. González‐González , and E. Ezcurra . 1995. “Patrones de Distribución Ficoflorística en el Sistema Lagunar de Nichupté, Quintana Roo, México.” Acta Botanica Mexicana 31: 19–32. 10.21829/abm31.1995.734.

[ece371826-bib-0020] Collado‐Vides, L. , J. González‐González , and M. Gold‐Morgan . 1994. “A Descriptive Approach to the Floating Masses of Algae of a Mexican Caribbean Coastal Lagoon.” Botanica Marina 37: 391–396. 10.1515/botm.1994.37.5.391.

[ece371826-bib-0021] del Carmen Merino‐Virgilio, F. , Y. B. Okolodkov , A. C. Aguilar‐Trujillo , and J. A. Herrera‐Silveira . 2013. “Phytoplankton of the Northern Coastal and Shelf Waters of the Yucatan Peninsula, Southeastern Gulf of Mexico, Mexico.” Check List 9, no. 4: 771–779. 10.15560/9.4.771.

[ece371826-bib-0022] Desikachary, T. V. 1959. “Cyanophyta.” In I.C.A.R. Monograph on Algae. 686 pp. Indian Council of Agricultural Research.

[ece371826-bib-0023] Dreckmann, K. M. , and G. De Lara‐Isassi . 2001. “Historia Taxonómica del Género *Gracilaria* Greville (Gracilariaceae, Rhodophyta).” Revista de la Sociedad Mexicana de Historia Natural 50: 33–48.

[ece371826-bib-0024] Drouet, F. 1968. “Revision of the Classification of the Oscillatoriaceae.” Proceedings of the Academy of Natural Sciences of Philadelphia Monograph 15: 1–370.

[ece371826-bib-0025] Drouet, F. 1973. Revision of the Nostocaceae With Cylindrical Trichomes (Formerly Scytonemataceae and Rivulariaceae). 292 pp. London & New York Press.

[ece371826-bib-0026] Drouet, F. 1978. “Revision of the Nostocaceae With Constricted Trichomes.” Beihefte zur Nova Hedwigia 57: i–v: 1–258.

[ece371826-bib-0027] Drouet, F. 1981. “Revision of the Stigonemataceae With a Summary of the Classification of the Blue‐Green Algae.” Beihefte zur Nova Hedwigia 66: 1–221.

[ece371826-bib-0028] Drouet, F. , and W. A. Daily . 1956. “Revision of the Coccoid Myxophyceae.” Butler University Botanical Studies 12, no. 1: 1–218.

[ece371826-bib-0135] Earle, SA. 1972. Phaeophyta of the eastern Gulf of Mexico. In Bushnell VC. (ed). Benthic algae and seagrasses, in Chemistry, primary productivity, and benthic marine algae of the Gulf of Mexico, Serial Atlas of the Marine Environment, 22 pp. 15–18. New York: American Geo‐ graphical Society, New York, USA.

[ece371826-bib-0030] Forti, A. 1907. Sylloge Algarum Omnium Hucusque Cognitarum. V [of Toni, J.B. “Sylloge Algarum Omnium”], 1–761. Sylloge Myxophycearum.

[ece371826-bib-0131] Fremy, P. 1939. “Cyanophyceae. In: F. BØrgesen, The Marine Algae of the Danish WestIndies.” Dansk Botanisk Arkiv 9, no. 7: 1–47.

[ece371826-bib-0031] Fritsch, F. E. 1945a. The Structure and Reproduction of the Algae. Volume II. Foreword, Phaeophyceae, Rhodophyceae, Myxophyceae, 1–939. Cambridge University Press.

[ece371826-bib-0032] Fritsch, F. E. 1945b. “Studies in the Comparative Morphology of the Algae IV. Algae and Archegoniate Plants.” Annals of Botany, New Series 9: 1–29.

[ece371826-bib-0033] Gärtner, G. 2018. “Checklist of Algae From Bulgarian Thermal Waters.” Annual of Sofia University “St. Kliment Ohridski”, Faculty of Biology, Book 2—Botany 102: 49–73.

[ece371826-bib-0034] Garza‐Barrientos, M. A. 1976. “Primeras Consideraciones Referentes a la Flora Marina del Sureste de la República Mexicana.” In Mem. I Reunión Latinoamericana Sobre Ciencia y Tecnología de los Océanos. Heroica Escuela Naval, Secretaría de Marina (Antón Lizardo, Veracruz, México, 1976), vol. 1, 210–239. Secretaria de Marina.

[ece371826-bib-0035] Geitler, L. 1925. “Beiträge zur Kenntnis der Flora Ostholsteinischer Seen.” Archiv für Protistenkunde 52: 603–611.

[ece371826-bib-0037] Geitler, L. 1932. “Cyanophyceae.” In Rabenhorts L. Kryptogamen‐Flora von Deutschland, Österreich und der Schweiz, vol. 14, 2nd ed., 673–1196. Akademische Verlagsgesellschaft.

[ece371826-bib-0038] Gomont, M. 1890. “Essai de Classification des Nostocacées Homocystées.” Journal de Botanique 4: 349–357.

[ece371826-bib-0039] Gomont, M. 1892. “Monographie des Oscillariées (Nostocacées Homocystées).” Annales Des Sciences Naturelles Botanique Série 715: 263–368.

[ece371826-bib-0040] González‐Resendiz, L. , J. R. Johansen , L. Alba‐Lois , et al. 2018. “ *Nunduva*, a New Marine Genus of Rivulariaceae (Nostocales, Cyanoprokaryote) From Marine Rocky Shores.” Fottea, Olomouc 18, no. 1: 86–105. 10.17129/botsci.3391.

[ece371826-bib-0041] González‐Resendiz, L. , J. R. Johansen , V. Escobar‐Sánchez , C. Segal‐Kischinevzky , L. F. Jiménez‐García , and H. León‐Tejera . 2018. “Two New Species of *Phyllonema* (Rivulariaceae, Cyanoprokaryote) With an Emendation of the Genus.” Journal of Phycology 54, no. 5: 638–652. 10.1111/jpy.12769. 30055049

[ece371826-bib-0042] González‐Resendiz, L. , J. R. Johansen , H. León‐Tejera , et al. 2019. “A Bridge Too Far in Naming Species: A Total Evidence Approach Does Not Support Recognition of Four Species in *Desertifilum* (Cyanoprokaryote).” Journal of Phycology 55: 898–911. 10.1111/jpy.12867. 31012104

[ece371826-bib-0043] González‐Resendiz, L. , H. León‐Tejera , J. Díaz‐Larrea , L. Alba‐Lois , and C. Segal‐Kischinevzky . 2013. “ *Hassallia Littoralis* sp. nov. (Cyanoprokaryote, Microchaetaceae) From Mexico's Marine Supralittoral Based on Morphological and Molecular Evidence.” Phytotaxa 137, no. 1: 35–47. 10.11646/phytotaxa.137.1.4.

[ece371826-bib-0045] González‐Reséndiz, M. L. , L. M. García‐Sánchez , J. G. Rodríguez‐Juárez , J. S. De Gyves‐López , and H. L. Tejera . 2014. “Caracterización de Ambientes Algales en Playa Muñecos, Veracruz, México.” Investigación Universitaria Multidisciplinaria 13, no. 13: 36–42.

[ece371826-bib-0046] Guiry, M. D. , and G. M. Guiry . 2025. AlgaeBase. World‐wide Electronic Publication, National University of Ireland. https://www.algaebase.org.

[ece371826-bib-0047] Günther, A. 1990. “Distribution and Bathymetric Zonation of Shell‐Boring Endoliths in Recent Reef and Shelf Environments: Cozumel, Yucatan (Mexico).” Facies 22, no. 1: 233–261.

[ece371826-bib-0049] Hauer, T. , and J. Komárek . 2024. CyanoDB 2.0—On‐Line Database of Cyanoprokaryotel Genera. World‐wide Electronic Publication, University of South Bohemia y Institute of Botany AS CR. http://www.cyanodb.cz.

[ece371826-bib-0050] Hernández‐Casas, C. M. , A. C. Mendoza‐González , L. E. Mateo‐Cid , and C. F. Vargas‐Mendoza . 2024. “Temporal Variation of Epiphytic Algae on *Digenea mexicana* (Rhodophyta: Ceramiales) in a Community Located in the South of Quintana Roo, México.” Regional Studies in Marine Science 72: 103433. 10.1016/j.rsma.2024.103433.

[ece371826-bib-0051] Herrera‐Silveira, J. A. 2006. “Lagunas Costeras de Yucatán (SE, México) Investigación, Diagnóstico y Manejo.” Ecotropicos 19, no. 2: 94–108.

[ece371826-bib-0052] Hoffmann, L. 1999. “Marine Cyanoprokaryote in Tropical Regions: Diversity and Ecology.” European Journal of Phycology 34: 371–379. 10.1080/09670269910001736432.

[ece371826-bib-0053] Hoffmann, L. , J. Komárek , and J. Kastovsky . 2005. “System of Cyanoprokaryotes (Cyanoprokaryote)—State in 2004.” Algological Studies (Cyanoprokaryotel Research 6) 117: 95–115. 10.1127/1864-1318/2005/0117-0095.

[ece371826-bib-0054] Hoiczyk, E. 1998. “Structural and Biochemical Analysis of the Sheath of *Phormidium uncinatum* .” Journal of Bacteriology 180, no. 15: 3923–3932. 9683490 10.1128/jb.180.15.3923-3932.1998 PMC107377

[ece371826-bib-0055] Hu, C. , and P. Rzymski . 2022. “Non‐Photosynthetic Melainabacteria (Cyanoprokaryote) in Human Gut: Characteristics and Association With Health.” Life 12, no. 4: 476. 10.3390/life12040476. 35454968 PMC9029806

[ece371826-bib-0056] Huerta, M. L. , and M. A. Garza‐Barrientos . 1980. “Contribución al Conocimiento de la Flora Marina de la Zona Sur del Litoral de Quintana Roo, México. (Contribution to marine flora research of the south littoral of Quintana Roo, Mexico).” Anales de la Escuela Nacional de Ciencias Biológicas 23: 25–44.

[ece371826-bib-0057] Huerta‐Múzquiz, L. 1958. “Contribución al Conocimiento de las Algas de los Bajos de la Sonda de Campeche, Cozumel e Isla Mujeres.” Anales de la Escuela Nacional de Ciencias Biológicas 9: 115–123.

[ece371826-bib-0058] Huerta‐Múzquiz, L. 1961. “Flora Marina de los Alrededores de la Isla Pérez, Arrecife Alacranes, Sonda de Campeche, México. Anales de la Escuela Nacional de Ciencias Biológicas.” México 10: 10–22.

[ece371826-bib-0059] Huerta‐Múzquiz, L. , A. C. Mendoza‐González , and L. E. Mateo‐Cid . 1987. “Avance Sobre un Estudio de las Algas Marinas de la Península de Yucatán.” Phytologia 62: 23–53.

[ece371826-bib-0060] Huerta‐Múzquiz, L. , M. E. Sánchez‐Rodríguez , and M. L. Chávez‐Barrera . 1977. Algas Marinas de Isla de Enmedio, Veracruz, 314–325. Memorias del V. Congreso Nacional de Oceanografía.

[ece371826-bib-0061] Humm, H. J. , and H. H. Hildebrand . 1962. “Marine Algae From the Gulf Coast of Texas and Mexico.” Publications of the Institute of Marine Sciences 8: 227–268.

[ece371826-bib-0062] Johansen, J. R. , L. González‐Resendiz , V. Escobar‐Sánchez , et al. 2021. “When Will Taxonomic Saturation Be Achieved? A Case Study in *Nunduva* and *Kyrtuthrix* (Rivulariaceae, Cyanoprokaryote).” Journal of Phycology 57, no. 6: 1699–1720. 10.1111/jpy.13201. 34289115

[ece371826-bib-0063] Kabirnataj, S. , G. A. Nematzadeh , A. F. Talebi , et al. 2020. “Description of Novel Species of *Aliinostoc, Desikacharya* and *Desmonostoc* Using a Polyphasic Approach.” International Journal of Systematic and Evolutionary Microbiology 70, no. 5: 3413–3426. 10.1099/ijsem.0.004188. 32375955

[ece371826-bib-0064] Kilgore, C. , J. R. Johansen , T. Mai , T. Hauer , D. A. Casamata , and C. Sheil . 2018. “Molecular Characterization of *Geitleria appalachiana sp. nov*. (Nostocales, Cyanoprokaryote) and Formation of Geitleriaceae *Fam. Nov* .” Faculty Bibliography 59: 150–166. 10.5507/fot.2018.002.

[ece371826-bib-0065] Kim, C. S. 1964. “Marine Algae of Alacran Reef, Southern Gulf of México.” PhD thesis. Duke University. 213 pp. https://www.proquest.com/openview/b9ac409608542342179ed70abeb96189/1.pdf?pq‐origsite=gscholar&cbl=18750&diss=y.

[ece371826-bib-0066] Komárek, J. 2010a. “Modern Taxonomic Revision of Planktic Nostocacean Cyanoprokaryote: A Short Review of Genera.” Hydrobiologia 639, no. 1: 231–243. 10.1007/s10750-009-0030-4.

[ece371826-bib-0067] Komárek, J. 2010b. “Recent Changes (2008) in Cyanoprokaryote Taxonomy Based on a Combination of Molecular Background With Phenotype and Ecological Consequences (Genus and Species Concept).” Hydrobiologia 639, no. 1: 245–259. 10.1007/s10750-009-0031-3.

[ece371826-bib-0068] Komárek, J. 2013. “Susswasserflora von Mitteleuropa. Freshwater Flora of Central Europe. 19/3.” In Cyanoprokaryota 3. Teil/3rd Part: Heterocytous Genera, edited by B. Budel , L. Krienitz , G. Y. Gartner , and M. Schagerl , 574 pp. Elsevier/Spektrum.

[ece371826-bib-0069] Komárek, J. , and K. Anagnostidis . 1986. “Modern Approach to the Classification System of Cyanophytes 2—*Chroococcales* .” Archiv für Hidrobiologie/Algological Studies 43: 157–226.

[ece371826-bib-0070] Komárek, J. , and K. Anagnostidis . 1989. “Modern Approach to the Classification System of Cyanophytes 4—Nostocales.” Algological Studies 56: 247–345.

[ece371826-bib-0071] Komárek, J. , and K. Anagnostidis . 1999. “Cyanoprokaryota 1. Chroococcales.” In Subwasserflora Con Mitteleuropa, edited by H. Ettl , G. Gärtner , H. Heynig , and D. Mollenhauer , 548 pp. Spektrum, Akademischer Verlag.

[ece371826-bib-0072] Komárek, J. , and K. Anagnostidis . 2005. Cyanoprokaryota 19 Teil/2nd Part: Oscillatoriales. Susswasserflora von Mitteleuropa. 19/2, edited by B. Budel , L. Krienitz , G. Gartner , and M. Schagerl , 759 pp. Elsevier/Spektrum, Heildelberg.

[ece371826-bib-0073] Komárek, J. , J. Kaštovský , J. Mares , and J. R. Johansen . 2014. “Taxonomic Classification of Cyanoprokaryotes (Cyanoprokaryotel Genera) 2014, Using a Polyphasic Approach.” Preslia 86: 295–335.

[ece371826-bib-0074] Kumar, N. , A. Saraf , S. Pal , D. Mishra , P. Singh , and J. R. Johansen . 2023. “Circumscription of *Fulbrightiella* gen. nov. and *Sherwoodiella* gen. nov., Two Novel Genera in the Calotrichaceae (Nostocales, Cyanoprokaryote).” Journal of Phycology 59, no. 1: 204–220. 10.1111/jpy.13297. 36331047

[ece371826-bib-0075] Kützing, F. T. 1847. Tabulae Phycologicae; Oder, Abbildungen der Tange. Nordhausen: Gedruckt Auf Kosten des Verfassers (in Commission Bei W. Köhne). Vol. 1, 3–5, 17–36. Nordhausen: Gedruckt Auf Kosten des Verfassers (in Commission Bei W. Köhne).

[ece371826-bib-0076] León‐Tejera, H. , E. Cabrera , L. González , A. García , B. Ramírez , and M. Peralta . 2019. Catálogo de Autoridades Taxonómicas de Cyanoprocaryota Marinos Bentónicos de México. Universidad Nacional Autónoma de México. Facultad de Ciencias. Informe final SNIB‐CONABIO, Proyecto No. KT016.

[ece371826-bib-0077] León‐Tejera, H. , M. Gold‐Morgan , and G. Montejano . 2009. “Benthic Cyanoprokaryota (Cyanoprokaryote) of the Gulf of Mexico.” In Gulf of Mexico Origin, Waters and Biota, edited by D. L. Felder and D. K. Camp , 47–56. Texas AyM University Press.

[ece371826-bib-0078] León‐Tejera, H. , L. González–Resendiz , J. R. Johansen , C. Segalkischinevzky , V. Escobar–Sánchez , and L. Alba–Lois . 2016. “Phylogenetic Position Reevaluation of *Kyrtuthrix* and Description of a New Species *K. Huatulcensis* From Mexico's Pacific Coast.” Phytotaxa 278, no. 1: 001–018. 10.11646/phytotaxa.278.1.1.

[ece371826-bib-0079] León‐Tejera, H. P. , E. Cabrera‐Becerril , and D. Parra‐Toriz . 2023. “Lista Taxonómica de las Especies de Cianobacterias bentónicas Marinas Con Distribución en México. Comisión Nacional Para el Conocimiento y Uso de la Biodiversidad. Checklist dataset.” Accessed via GBIF.org on June 5, 2023. 10.15468/mmez37.

[ece371826-bib-0080] León‐Tejera, H. P. , L. González‐Resendiz , E. Cabrera‐Becerril , et al. 2016. “Estado Del Conocimiento de Cianoprocariontes Bénticos Marinos de La Costa Atlántica Mexicana.” Responsabilidad Para la Sustentabilidad de la Zona Costera. Número Especial 4. Año 6, vol. 11, 95–105.

[ece371826-bib-0081] Lind, O. , L. Dávalos‐Lind , C. López , M. López , and J. D. Bressie . 2016. “Seasonal Morphological Variability in an *In Situ* Cyanoprokaryote Monoculture: Example From a Persistent *Cylindrospermopsis* Bloom in Lake Catemaco, Veracruz, Mexico.” Journal of Limnology 75: 66–80.

[ece371826-bib-0082] Martínez‐Arroyo, A. , S. Abundes , M. E. González , and I. Rosas . 2000. “On the Influence of Hot‐Water Discharges on Phytoplankton Communities From a Coastal Zone of the Gulf of Mexico.” Water, Air, and Soil Pollution 119: 209–230. 10.1023/A:1005161309609.

[ece371826-bib-0083] Martínez‐Lozano, S. , and O. Guajardo‐Ríos . 1991. “Lista Sistemática de las Algas Marinas del Puerto El Mezquital, Matamoros, Tamaulipas, México.” BIOTAM. Universidad Autónoma de Tamaulipas 3, no. 3: 16–26.

[ece371826-bib-0086] Mateo‐Cid, L. E. , and A. C. Mendoza‐González . 1991. “Algas Marinas Bénticas de la Isla Cozumel, Quintana Roo, México.” Acta Botanica Mexicana 16: 57–87. 10.21829/abm16.1991.626.

[ece371826-bib-0087] Mateo‐Cid, L. E. , A. C. Mendoza‐González , G. Ávila Ortiz , and S. Díaz Martínez . 2013. “Algas Marinas Bentónicas del litoral de Campeche, México.” Acta Botanica Mexicana 104: 53–92. 10.21829/abm104.2013.57.

[ece371826-bib-0088] Mateo‐Cid, L. E. , A. C. Mendoza‐González , and S. Fredericq . 2013. “A Checklist of Subtidal Seaweeds From Campeche Banks, Mexico.” Acta Botánica Venezuelica 36, no. 2: 95–108.

[ece371826-bib-0089] Mateo‐Cid, L. E. , A. C. Mendoza‐González , and C. Galicia‐García . 1996. “Algas Marinas de Isla Verde, Veracruz, México.” Acta Botanica Mexicana 36: 59–75. 10.21829/abm36.1996.762.

[ece371826-bib-0090] McNeill, J. , F. R. Barrie , W. R. Buck , et al., eds. 2006. International Code of Botanical Nomenclarure. A.R.G. Gantner Verlag KG 568 pp.

[ece371826-bib-0091] Mendoza‐González, A. C. , and L. E. Mateo‐Cid . 1985. “Contribución al Estudio Florístico‐Ficológico de la Costa Occidental de Baja California.” Phytologia 59: 17–33.

[ece371826-bib-0092] Mendoza‐González, A. C. , and L. E. Mateo‐Cid . 1992. “Algas Marinas Bentónicas de Isla Mujeres, Quintana Roo, México.” Acta Botanica Mexicana 19: 37–61. 10.21829/abm19.1992.646.

[ece371826-bib-0093] Mendoza‐González, A. C. , L. E. Mateo‐Cid , J. A. Acosta‐Calderón , A. Vázquez‐Rodríguez , C. M. Hernández‐Casas , and G. A. Garduño‐Acosta . 2016. “Marine Seaweeds of the Yucatán Peninsula: Diversity, Economic Importance, and Conservation.” In Marine Benthos: Biology, Ecosystem Functions and Environmental Impact, edited by R. Riosmena‐Rodríguez , 39–83. Hauppauge.

[ece371826-bib-0095] Mendoza‐González, A. C. , L. E. Mateo‐Cid , and D. Y. García‐López . 2017. “Inventory of Benthic Marine and Estuarine Algae and Cyanoprokaryote for Tabasco, Mexico.” Biota Neotropica 17, no. 4: e20170379. 10.1590/1676-0611-bn-2017-0379.

[ece371826-bib-0096] Mendoza‐González, A. C. , L. E. Mateo‐Cid , and R. B. Searles . 2007. “Yucatán Seaweeds From the Offshore Waters of Isla Mujeres, Quintana Roo, Mexico.” Botanica Marina 50, no. 5–6: 280–287. 10.1515/BOT.2007.032.

[ece371826-bib-0097] Nava‐Olvera, R. , L. E. Mateo‐Cid , I. González‐Contreras , and A. C. Mendoza‐González . 2024. “Spatio‐Temporal Variation in Cyanobacteria and Epiphytic Algae of *Thalassia testudinum* in Two Localities of Southern Quintana Roo, Mexico.” Diversity 16, no. 6: 321. 10.3390/d16060321.

[ece371826-bib-0098] Nava‐Olvera, R. , L. E. Mateo‐Cid , A. C. Mendoza‐González , and D. Y. García‐López . 2017. “Macroalgas, Microalgas y Cianobacterias Epífitas Del Pasto Marino *Thalassia testudinum* (Tracheophyta: Alismatales) en Veracruz y Quintana Roo, Atlántico Mexicano.” Revista de Biología Marina y Oceanografía 52, no. 3: 429–439. 10.4067/S0718-19572017000300002.

[ece371826-bib-0099] Nguyen, X. V. , X. T. Nguyen , R. P. Kittle‐Iii , and K. J. Mcdermid . 2022. “ *Meristotheca lysonensis* sp. nov. (Solieriaceae, Rhodophyta), a New Flattened Species From Vietnamese Waters.” Phytotaxa 574, no. 2: 137–148. 10.11646/phytotaxa.574.2.2.

[ece371826-bib-0100] Novelo, E. , and R. Tavera . 2019. bdLACET Base de Datos de Algas Continentales. Facultad de Ciencias, UNAM. https://bdlacet.mx.

[ece371826-bib-0101] Oren, A. , J. Mareš , and R. Rippka . 2022. “Validation of the Names *Cyanobacterium* and *Cyanobacterium stanieri*, and Proposal of Cyanobacteriota Phyl. Nov.” International Journal of Systematic and Evolutionary Microbiology 72, no. 10: 005528. 10.1099/ijsem.0.005528. 36251754

[ece371826-bib-0102] Ortega, M. M. 1995. “Observaciones del Fitobentos de la Laguna de Términos, Campeche, México.” Anales del Instituto de Biología Serie Botánica 66, no. 1: 1–36.

[ece371826-bib-0103] Ortega, M. M. , J. L. Godínez , and G. Garduño‐Solórzano . 2001. Catálogo de Algas Bénticas de Las Costas Mexicanas del Golfo de México y Mar Caribe. 594 pp. Instituto de Biología, Universidad Nacional Autónoma de México.

[ece371826-bib-0104] Ortegón‐Aznar, I. , and H. León‐Tejera . 2022. “Diversidad de Macroalgas y Cianoprocariontes Marinos de la Costa Norte de la Península de Yucatán, México.” Hidrobiológica 32, no. 3: 309–317. 10.24275/uam/izt/dcbs/hidro/2022v32n3/Ortegon.

[ece371826-bib-0105] Ortegón‐Aznar, I. , H. León‐Tejera , M. Gold‐Morgan , and N. Ramírez‐Miss . 2008. “Preliminary Results on Marine Algae of Madagascar Reef, Yucatan, México: A Functional Group Approach.” In Proceedings of the 11th International Coral Reef Symposium, 1373–1376.

[ece371826-bib-0106] Pedroche, F. F. 2022. “La Obra de J. Agardh: Algae Liebmanniae, Revisitada 175 Años Después.” Hidrobiológica 32, no. 3: 171–182. 10.24275/uam/izt/dcbs/hidro/2022v32n3/Pedroche.

[ece371826-bib-0107] Poot‐Delgado, C. A. , and Y. Guzmán Noz . 2009. “Composición y Abundancia del Fitoplancton Marino, Con Énfasis en las Especies Potencialmente Tóxicas y/o Nocivas, en la Bahía de Campeche, México.” Resúmenes III Taller Sobre Florecimientos Algales Nocivos, Acapulco, 38.

[ece371826-bib-0108] Poot‐Delgado, C. A. , Y. Okolodkov , J. A. Aké‐Castillo , and J. R. Von Osten . 2017. “Cianobacterias Potencialmente Nocivas en los Bancos Ostrícolas de la Laguna de Términos, Sureste del Golfo de México.” Acta Biológica Colombiana 23, no. 1: 51–58. 10.15446/abc.v23n1.65809.

[ece371826-bib-0109] Poot‐Delgado, C. A. , Y. B. Okolodkov , J. A. Aké‐Castillo , and J. R. Von Osten . 2015. “Fitoplancton Potencialmente Nocivo en el Muelle la Puntilla, Laguna de Términos, Sureste del Golfo de México.” Biocyt: Biología, Ciencia y Tecnología 8, no. 29: 570–582.

[ece371826-bib-0110] Rabenhorst, L. 1865. Flora Europaea Algarum Acquae Dulcis et Submarinae Auctore Ludovico Rabenhorst: Algas Phycochromaceas Complectens, 2. E. Kummerum. 10.5962/bhl.title.7029.

[ece371826-bib-0111] Ramírez‐Rodríguez, A. , R. Blanco Pérez , and Y. D. Okolodkov . 2011. “Diversidad de Algas Epifitas Marinas.” In Comisión Nacional para el Conocimiento y Uso de la Biodiversidad (Conabio). La biodiversidad en Veracruz: Estudio de Estado. Vol. II. Diversidad de especies: Conocimiento actual, edited by A. Cruz‐Angon , 59–69. Universidad Veracruzana, Instituto de Ecología, A.C.

[ece371826-bib-0112] Rippka, R. , J. Deruelles , J. B. Waterbury , M. Herdman , and R. Y. Stanier . 1979. “Generic Assignment, Strain Histories and Properties of Pure Cultures of Cyanoprokaryote.” Journal of General Microbiology 111: 1–61. 10.1099/00221287-111-1-1.

[ece371826-bib-0113] Sánchez‐Rodríguez, M. E. 1980. “Ficoflora del Sustrato Rocoso Dentro de las Costas del Golfo de México, México.” Boletim do Instituto Oceanográfico 29: 347–350. 10.1590/S1679-87591980000200069.

[ece371826-bib-0114] Saraf, A. , A. Suradkar , H. G. Dawda , et al. 2019. “Phylogenetic Complexities of the Members of Rivulariaceae With the Re‐Creation of the Family Calotrichaceae and Description of *Dulcicalothrix necridiiformans* Gen Nov., sp Nov., and Reclassification of *Calothrix desertica* .” FEMS Microbiology Letters 366, no. 17: fnz219. 10.1093/femsle/fnz219. 31633749

[ece371826-bib-0132] Schaffner, J. H. 1922. “The Classification of Plants. XII.” Ohio Journal of Science 22, no. 129: 39.

[ece371826-bib-0116] Silva, P. C. , P. W. Basson , and R. L. Moe . 1996. Catalogue of the Benthic Marine Algae of the Indian Ocean, 79:1–1259. University of California Publications in Botany.

[ece371826-bib-0117] Silva‐Pineda, A. 1977. “ *Goniolina geométric*a (Chlorophyta‐Dasycladaceae) de la Formación San Ricardo (Jurásico Superior) del Estado de Chiapas.” Revista Mexicana de Ciencias Geológicas 1, no. 1: 64–68.

[ece371826-bib-0118] Šmarda, J. , D. Šmajs , J. Komrska , and V. Krzyžánek . 2002. “S‐Layers on Cell Walls of Cyanobacteria.” Micron 33, no. 3: 257–277. 11742749 10.1016/s0968-4328(01)00031-2

[ece371826-bib-0119] Smith, G. M. 1950. The Fresh‐Water Algae of the United States. 2nd ed, 1–719. McGraw‐Hill Book Company.

[ece371826-bib-0120] Soto, L. A. , A. Estradas , R. Herrera , et al. 2004. “Biodiversidad Marina en la Sonda de Campeche.” In PEMEX y la Salud Ambiental de la Sonda de Campeche, edited by L. A. Soto and M. C. González‐Macías , 205–238. Instituto Mexicano del Petróleo.

[ece371826-bib-0121] Stanier, R. Y. , W. R. Sistrom , T. A. Hansen , et al. 1978. “Proposal to Place the Nomenclature of the Cyanoprokaryote (Blue‐Green Algae) Under the Rules of the International Code of Nomenclature of Bacteria.” International Journal of Systematic and Evolutionary Microbiology 28, no. 2: 335–336. 10.1099/00207713-28-2-335.

[ece371826-bib-0122] Strunecký, O. , A. P. Ivanova , and J. Mareš . 2023. “An Updated Classification of Cyanoprokaryotel Orders and Families Based on Phylogenomic and Polyphasic Analysis.” Journal of Phycology 59, no. 1: 12–51. 10.1111/jpy.13304. 36443823

[ece371826-bib-0123] Taylor, W. R. 1928. The Marine Algae of Florida, with Special Reference to the Dry Tortugas. Carnegie Institution of Washington Publication 379. Vol. 25. Papo Tortugas Laboratory 219 pp.

[ece371826-bib-0124] Tunnell, J. W., Jr. , E. A. Chávez , and K. Wither . 2007. Coral Reefs of the Southern Gulf of Mexico, 256 Pp. Texas A&M University Press.

[ece371826-bib-0125] Vaccarino, M. A. , and J. R. Johansen . 2012. “ *Brasilonema Angustatum* sp. Nov. (Nostocales), A New Filamentous Cyanoprokaryotel Species From the Hawaiian Islands 1.” Journal of Phycology 48, no. 5: 1178–1186. 10.1111/j.1529-8817.2012.01203.x. 27011277

[ece371826-bib-0126] van Tussenbroek, B. I. , and L. Collado Vides . 2000. “Filamentous Algae Dominate a Tropical Reef Community in the Mexican Caribbean: An Unexpected Organization of Reef Vegetation.” Botanica Marina 43, no. 6: 547–557. 10.1515/BOT.2000.055.

[ece371826-bib-0127] Vodenicharov, D. 1971. Flora of Bulgaria. Algae, 642 pp, edited by S. Draganov and D. Temniskova . Sofia, Narodna Prosveta.

[ece371826-bib-0128] Whitton, B. A. , and M. Potts . 2012. “Introduction to the Cyanoprokaryote.” In Ecology of Cyanoprokaryote II. Their Diversity in Space and Time. 700 pp, edited by B. A. Whitton . Springer. 10.1007/978-94-007-3855-3.

[ece371826-bib-0129] Wood, H. C. 1868. “Prodromus of a Study of the Freshwater Algae of North America.” Proceedings of the American Philosophical Society 11: 119–145.

