# Supplementary material for: Taxonomic History and State of Knowledge of the Marine Species in Nostocales (Cyanoprokaryote) From the Mexican Atlantic
Source: Ecol Evol. 2025 Jul 30;15(8):e71826. doi: 10.1002/ece3.71826 (PMC12310826; doi:10.1002/ece3.71826)
Supplement: Supplementary file 1 — Appendix S1. [file ECE3-15-e71826-s001.docx]

- **Annex 1. Classification system of Nostocales Cyanoprokaryotes according to Burnett & Flahault, 1886-1887**

| **Order** | **Sub order** | **Family** | **Sub Family** | **Tribu** | **Subtribu 1** | **Sectio** | **Genera** | **Especies** |
| --- | --- | --- | --- | --- | --- | --- | --- | --- |
| **I.Schizophyceae** | **II. Phycochromophyceae** | **I. Hormogoneae** | **I. eterocysteae** | Tribu I. Rivulariaceae | 1. Leptochaetae |  | *Leptochaete* | *L. crustacea* |
|  |  |  |  |  |  |  |  | *L.fonticola* |
|  |  |  |  |  |  |  |  | *L. parasitica.* |
|  |  |  |  |  |  |  | *Anphitrhix* | *A. janthina* |
|  |  |  |  |  |  |  |  | *A. violacea* |
|  |  |  |  |  | II. Mastichotricheae | I. Homoeothrix | *Calothrix* | *C. rubra* |
|  |  |  |  |  |  |  |  | *C. balearica* |
|  |  |  |  |  |  |  |  | *C. juliana* |
|  |  |  |  |  |  | II. Eucalothrix | *Calothrix* | *C. confervicola* |
|  |  |  |  |  |  |  |  | *C. consociata* |
|  |  |  |  |  |  |  |  | *C. fusco-violacea* |
|  |  |  |  |  |  |  |  | *C. scopolorum* |
|  |  |  |  |  |  |  |  | *C. contarenii* |
|  |  |  |  |  |  |  |  | *C. pulvinata* |
|  |  |  |  |  |  |  |  | *C. paratica* |
|  |  |  |  |  |  |  |  | *C. aeruginea* |
|  |  |  |  |  |  |  |  | *C. crustacea* |
|  |  |  |  |  |  |  |  | *C. prolifera* |
|  |  |  |  |  |  |  |  | *C. fasciculata* |
|  |  |  |  |  |  |  |  | *C. vivipara* |
|  |  |  |  |  |  |  |  | *C. pilosa* |
|  |  |  |  |  |  |  |  | *C. fusca* |
|  |  |  |  |  |  |  |  | *C. stellaris* |
|  |  |  |  |  |  |  |  | *C. adscendens* |
|  |  |  |  |  |  |  |  | *C braunii* |
|  |  |  |  |  |  |  |  | *C. thermalos* |
|  |  |  |  |  |  |  |  | *C. castelii* |
|  |  |  |  |  |  |  | *Dichothrix* | *D. nordstedtii* |
|  |  |  |  |  |  |  |  | *D. olivacea* |
|  |  |  |  |  |  |  |  | *D. baueriana* |
|  |  |  |  |  |  |  |  | *D. orsiniana* |
|  |  |  |  |  |  |  |  | *D. gypsophila* |
|  |  |  |  |  |  |  |  | *D. compacta* |
|  |  |  |  |  |  |  |  | *D. fucicola* |
|  |  |  |  |  |  |  |  | *D. penicillata* |
|  |  |  |  |  |  |  | *Polythrix* | *P. corymbosa* |
|  |  |  |  |  |  |  | *Sacconema* | *S. rupestre* |
|  |  |  |  |  | III. Rivularieae | Eurivulariaceae | *Isactis* | *I. Plana* |
|  |  |  |  |  |  |  | *Rivularia* | *R. dura* |
|  |  |  |  |  |  |  |  | *R. minutula* |
|  |  |  |  |  |  |  |  | *R. rufescens* |
|  |  |  |  |  |  |  |  | *R. haematites* |
|  |  |  |  |  |  |  |  | *R. biasolettiana* |
|  |  |  |  |  |  |  |  | *R. atra* |
|  |  |  |  |  |  |  |  | *R. beccariana* |
|  |  |  |  |  |  |  |  | *R. vierllardi* |
|  |  |  |  |  |  |  |  | *R. nitida* |
|  |  |  |  |  |  |  |  | *R. bullata* |
|  |  |  |  |  |  |  |  | *R. mesenterica* |
|  |  |  |  |  |  |  |  | *R. polyotis* |
|  |  |  |  |  |  |  |  | *R. Australis* |
|  |  |  |  |  |  |  | *Gloeothricia* | *G. pisum* |
|  |  |  |  |  |  |  |  | *G. raberhorstii* |
|  |  |  |  |  |  |  |  | *G. salina* |
|  |  |  |  |  |  |  |  | *G. punctulata* |
|  |  |  |  |  |  |  |  | *G. natans* |
|  |  |  |  |  |  |  | *Brachytrichia* | *B. balani* |
|  |  |  |  |  |  |  |  | *B. quoyi* |
|  |  |  |  | Tribu II: Sirosiphoniaceae | I. Stigonemeae |  | *XI. Mastigocoleus* | *M. testarum* |
|  |  |  |  |  |  |  | *XII. Hapalosiphon* | *H. laminosus* |
|  |  |  |  |  |  |  | *XII. Hapalosiphon* | *H. pumilus* |
|  |  |  |  |  |  | Sub gen: *Fischerella* | *XIII. Stigonema* | *S. thermale* |
|  |  |  |  |  |  |  |  | *S. muscicola* |
|  |  |  |  |  |  |  |  | *S. tenue* |
|  |  |  |  |  |  | Sub Gen. II: *Sirosiphon* | *XIII. Stigonema* | *S. hormoides* |
|  |  |  |  |  |  |  |  | *S. oscellatum* |
|  |  |  |  |  |  |  |  | *S. panniforme* |
|  |  |  |  |  |  |  |  | *S. minutum* |
|  |  |  |  |  |  |  |  | *S. turfaceum* |
|  |  |  |  |  |  |  |  | *S. boliviense* |
|  |  |  |  |  |  |  |  | *S. informe* |
|  |  |  |  |  |  |  |  | *S. mamillosum* |
|  |  |  |  |  |  |  |  | *S. leprieurii* |
|  |  |  |  |  | II.Nostochopsideae |  | *XIV. Capsosira* | *C. brebissonii* |
|  |  |  |  |  |  |  | *XV. Nostochopsis* | *N. lobatus* |
|  |  |  |  | Tribu III: Scytonemaceae |  |  | *XVI. Microchaete* | *Mi. tenera* |
|  |  |  |  |  |  |  |  | *Mi. diplosiphon* |
|  |  |  |  |  |  |  |  | *Mi. grisea* |
|  |  |  |  |  |  |  |  | *Mi. vitiensis* |
|  |  |  |  |  |  | I. *Euscytonema* (Aquaticae) | *XVII. Scytonema* | *S. cincinnatum* |
|  |  |  |  |  |  |  |  | *S. polycystum* |
|  |  |  |  |  |  |  |  | *S. coatile* |
|  |  |  |  |  |  |  |  | *S. rivulare* |
|  |  |  |  |  |  |  |  | *S. arcangelii* |
|  |  |  |  |  |  | I.*Euscytonema* (terrestrial) |  | *S. stuposum* |
|  |  |  |  |  |  |  |  | *S. millei* |
|  |  |  |  |  |  |  |  | *S. guyanense* |
|  |  |  |  |  |  |  |  | *S. javanicum* |
|  |  |  |  |  |  |  |  | *S. ocellatum* |
|  |  |  |  |  |  |  |  | *S. varium* |
|  |  |  |  |  |  |  |  | *S. hofmanni* |
|  |  |  |  |  |  |  |  | *S. ambigum* |
|  |  |  |  |  |  | II. *Myochrotes* |  | *S. tolypotrichoides* |
|  |  |  |  |  |  |  |  | *S. flavo-viride* |
|  |  |  |  |  |  |  |  | *S. figuratum* |
|  |  |  |  |  |  |  |  | *S. myochrous* |
|  |  |  |  |  |  | III. *Petalonema* |  | *S. crsutaceum* |
|  |  |  |  |  |  |  |  | *S. velutinum* |
|  |  |  |  |  |  |  |  | *S. involvens* |
|  |  |  |  |  |  |  |  | *S. crassum* |
|  |  |  |  |  |  |  |  | *S. densum* |
|  |  |  |  |  |  |  |  | *S. alatum* |
|  |  |  |  |  |  |  | *XVIII. Hassalia* | *H. bouteillei* |
|  |  |  |  |  |  |  |  | *H. byssoidea* |
|  |  |  |  |  |  |  | *XIX. Tolypothrix* | *T. distorta* |
|  |  |  |  |  |  |  |  | *T. lanata* |
|  |  |  |  |  |  |  |  | *T. tenuis* |
|  |  |  |  |  |  |  |  | *T. penicillata* |
|  |  |  |  |  |  |  |  | *T. limata* |
|  |  |  |  |  |  |  |  | *T. conglutinata* |
|  |  |  |  |  |  |  | *XX. Desmonema* | *D. wrangelii* |
|  |  |  |  |  |  |  |  | *D. floccosum* |
|  |  |  |  |  |  |  | *XXI. Hydrocoryne* | *H. spongiosa* |
|  |  |  |  |  |  |  | *XXII. Diplocolon* | *Di. Heppii* |
|  |  |  |  | Tribu IV: Nostoceae | I. Anabaeneae | I. Cuticularia | *XXIII. Nostoc* | *N. cuticulare* |
|  |  |  |  |  |  |  |  | *N. maculiforme* |
|  |  |  |  |  |  | II. Amorpha | *XXIII. Nostoc* | *N. hederulae* |
|  |  |  |  |  |  | III. Paludosa | *XXIII. Nostoc* | *N. entophytum* |
|  |  |  |  |  |  |  |  | *N. paludosum* |
|  |  |  |  |  |  | IV. Intricata | *XXIII. Nostoc* | *N. Linckia* |
|  |  |  |  |  |  |  |  | *N. piscinale* |
|  |  |  |  |  |  |  |  | *N. rivulare* |
|  |  |  |  |  |  |  |  | *N. carneum* |
|  |  |  |  |  |  |  |  | *N. spungifomre* |
|  |  |  |  |  |  | V. Humifusa | *XXIII. Nostoc* | *N: ellipsosporum* |
|  |  |  |  |  |  |  |  | *N. gelatinosum* |
|  |  |  |  |  |  |  |  | *N. passerinianum* |
|  |  |  |  |  |  |  |  | *N. muscorum* |
|  |  |  |  |  |  |  |  | *N. lumifusum* |
|  |  |  |  |  |  |  |  | *N.calciola* |
|  |  |  |  |  |  | VI. Comunnia | *XXIII. Nostoc* | *N. foliaceum* |
|  |  |  |  |  |  |  |  | *N. commune* |
|  |  |  |  |  |  |  |  | *N.sphaericum* |
|  |  |  |  |  |  |  |  | *N. minutum* |
|  |  |  |  |  |  | VII. Pruniformia | *XXIII. Nostoc* | *N. macrosporum* |
|  |  |  |  |  |  |  |  | *N. microscopicum* |
|  |  |  |  |  |  |  |  | *N. sphaeroides* |
|  |  |  |  |  |  |  |  | *N. caeruleum* |
|  |  |  |  |  |  |  |  | *N. edule* |
|  |  |  |  |  |  |  |  | *N. pruniforme* |
|  |  |  |  |  |  | VIII. Verrucosa | *XXIII. Nostoc* | *N. verrucosum* |
|  |  |  |  |  |  |  |  | *N. parmelioides* |
|  |  |  |  |  |  | IX. Zetterstedtiana | *XXIII. Nostoc* | *N. zertterstedtii* |
|  |  |  |  |  |  |  | *XXIV. Wollea* | *W. seccata* |
|  |  |  |  |  |  | I. *Trichormus* | *XXV. Anabaena* | *A. variabilia* |
|  |  |  |  |  |  |  |  | *A. hallensis* |
|  |  |  |  |  |  |  |  | *A. sphaerica* |
|  |  |  |  |  |  | II: *Dolichospermum* | *XXV. Anabaena* | *A. flos-aquae* |
|  |  |  |  |  |  |  |  | *A. circinalis* |
|  |  |  |  |  |  |  |  | *A. Ineaequalis* |
|  |  |  |  |  |  |  |  | *A. catenula* |
|  |  |  |  |  |  |  |  | *A. felisii* |
|  |  |  |  |  |  |  |  | *A. laxa* |
|  |  |  |  |  |  | III. *Sphaerozyga* | *XXV. Anabaena* | *A. oscillarioides* |
|  |  |  |  |  |  |  |  | *A. torulosa* |
|  |  |  |  |  |  |  | *XXVI. Aphanizomenon* | *Ap. Flos-aquae* |
|  |  |  |  |  |  |  |  | *Ap. Incurvum* |
|  |  |  |  |  |  |  | *XXVII. Nodularia* | *N. harveyana* |
|  |  |  |  |  |  |  |  | *N. sphaerocarpa* |
|  |  |  |  |  |  |  |  | *N. armorica* |
|  |  |  |  |  |  |  |  | *N. spumigena* |
|  |  |  |  |  |  |  | *XXVIII. Cylindrospermium* | *C. stagnale* |
|  |  |  |  |  |  |  |  | *C. licheniforme* |
|  |  |  |  |  |  |  |  | *C. muscicola* |
|  |  |  |  |  |  |  |  | *C. catenatum* |
|  |  |  |  |  | II. Aulosierae |  | *XXIX.Aulosira* | *Au. Laxa* |
|  |  |  |  |  |  |  |  | *Au. Implexa* |
|  |  |  |  |  |  |  | *XXX. Hormothamnion* | *H. solutum* |
|  |  |  |  |  |  |  |  | *H. enteromorphoides* |

- **Annex 2. Classification of the Nostocales order in Druet 1973**

| **Order** | **Family** | **Acepted Specie** | **Synonym** |
| --- | --- | --- | --- |
| **Hormogonales** | **Nostocaceae** | *Anabaina licheniformis* Bory de Saint-Vincent | *Cylindrospermum majus* Kützing |
|  |  | *Anabaina oscillarioides* Bory de Saint-Vincent | *Anabaina ambigua* C.B.Rao |
|  |  |  | *Anabaina indica Zeller* |
|  |  |  | *Anabaina pseudoscillatoria* Bory de Saint-Vincent |
|  |  |  | *Hormothamnium enteromorphoides* Grunow |
|  |  |  | *Hormothamnium solutum* Bornet & Grunow |
|  |  |  | *Komvophoron constrictum* (Szafer) Anagnostidis & Komárek |
|  |  |  | *Trichormus variabilis* (Kützing) Komárek & Anagnostidis |
|  |  | *Calothrix crustacea* Thuret | *Brachytrichia dalmatica* (Ercegovié) Frémy |
|  |  |  | *Brachytrichia maculans* Gomont |
|  |  |  | *Calothrix aeruginea* (Kützing) Thuret |
|  |  |  | *Calothrix confervicola* (Dillwyn) C. Agardh |
|  |  |  | *Calothrix contarenii* (Zanardini) Bornet & Flahault |
|  |  |  | *Calothrix fuscoviolacea* P. Crouan & H. Crouan |
|  |  |  | *Calothrix parasítica* (Chauvin) Thuret |
|  |  |  | *Calothrix pulvinata* (Mertens) C. Agardh |
|  |  |  | *Calothrix scopulorum* (Weber & Mohr) C. Agardh |
|  |  |  | *Gardnerula corymbosa* De Toni |
|  |  |  | *Gardnerula spongiosa* (Zanardini) Tseng & Hua |
|  |  |  | *Microchaete aeruginea* Batters |
|  |  |  | *Microchaete grisea* Thuret |
|  |  |  | *Microchaete vitiensis Askenasy ex Bornet & Flahault* |
|  |  |  | *Rivularia bullata Berkeley ex Bornet & Flahault* |
|  |  |  | *Rivularia polyotis* (J. Agardh) Hauck |
|  |  |  | *Scytonema endolithicum* Ercegovic |
|  |  |  | *Symploca hydnoides* Kützing ex Gomont |
|  |  |  | *Trichocladia nostocoides* Zanardini |
|  |  | *Calothrix parietina Thuret ex Bornet & Flahault* | *Calothrix marchica Lemmermann 1914* |
|  |  |  | *Dichothrix baueriana Bornet & Flahault* |
|  |  |  | *Dichothrix spiralis* Fritsch now) Bornet & Flahault |
|  |  |  | *Homoeothrix juliana* (Bornet & Flahault ex Gomont) Kirchner |
|  |  |  | *Scytonema rhizophorae Zeller, nom. inval. 1873* |
|  |  | *Nostoc commune* Vaucher | *Anabaina circinalis* Rabenhorst |
|  |  |  | *Anabaina spirioides* Klebahn |
|  |  |  | *Nostoc maculiforme* Bornet & Flahault |
|  |  | *Nostoc spumigena* (Mertens) Drouet | *Nodularia harveyana* (Thwaites) Thuret |
|  |  |  | *Nodularia spumigena* Mertens |
|  |  |  | *Nodularia spumigena* Mertens var. *major* (Kützing) Bornet & Flahault |
|  |  | *Scytonema hofmannii* C. Agardh | *Calothrix pilosa* Harvey |
|  |  |  | *Lyngbya crispa* (C. Agardh) C. Agardh |
|  |  |  | *Microchaete tenera* Thuret ex Bomet |
|  |  |  | *Scytonema guyanense* (Montagne) Bornet & Flahault |
|  |  |  | *Scytonema hofman-bangii*  C. Agardh |
|  |  |  | *Scytonema saleyeriense*  Weber-van Bosse |
|  |  |  | *Scytonema seagriefianum* Welsh |
|  |  |  | *Scytonema siculum* Borzi |
|  |  |  | *Tolypothrix byssoidea* (C. Agardh) Kirchner |
|  | **Stigonemataceae** | *Brachytrichia quoyi* (C. Agardh) Bornet & Flahault . | *Brachytrichia lloydii* (P. Crouan & H. Crouan) P. Silva |
|  |  | *Mastigocoleus testarum* Lagerheim |  |

- **Annex 3. Classification of cyanoprokaryota of the order Nostocales in the bacterial classification system according to Rippka *et al.* (1979). Synonyms = Strains registered under other names.**

| **Order or Section** | **Genera** | **Specie** | **Synonym** | **Strain** |
| --- | --- | --- | --- | --- |
| IV. Nostocales (36 strains 6 genera) | *Anabaena* | *Anabena variabilis* | *Cylindrospermium* sp. | ATCC 2921 1, ATCC 27892 |
|  |  | *Anabaena cylindrica* |  | ATCC 27899 |
|  |  | *Anabaena* sp. | *Nostoc moscorum* | ATCC 27893 |
|  |  | *Anabaena* sp. | *Nostoc* sp. | ATCC 27898 |
|  |  | *Anabaena* sp. |  | ATCC 29208, ATCC 29151 |
|  | *Nodularia* | *Nodularia* sp. |  | ATCC 29167 |
|  | *Cylindrospermium* | *Cylindrospermium stagnale* |  | ATCC 29535 PCC 73 10 1. |
|  |  | *Cylindrospermium maius* |  | ATCC 33001 PCC 7604. |
|  |  | *Cylindrospermium* sp. |  | ATCC 29204 PCC 7417 |
|  | *Nostoc* | *Nostoc* sp. | *Anabaena* sp. | ATCC 27897 |
|  |  | *Nostoc muscorum* |  | ATCC 27904, ATCC 29105 |
|  |  | *Nostoc* sp. | *Anabaena spiroides* | ATCC 27896 |
|  |  | *Nostoc* sp. |  | ATCC 29131, ATCC 29150, ATCC 29133, ATCC 29107, ATCC 29132, ATCC 29168, ATCC 2941 1 |
|  |  | *Nostoc* sp. | *Anabaenopsis circularis* | ATCC 27895 |
|  |  | *Nostoc* sp. | *Cylindrospermium licheniforme* | ATCC 29106 |
|  | *Scytonema* | *Scytonema* sp. |  | ATCC 29171 PCC 7110. |
|  | *Calothrix* | *Calothrix parietina* |  | ATCC 29156 |
|  |  | *Calothrix* sp. | *Tolypothrix tenuis* | ATCC 27914 |
|  |  | *Calothrix desertica* |  | ATCC 27901 |
|  |  | *Calothrix* sp. | *Nodularia sphaerocarpa* | ATCC 27905 |
|  |  | *Calothrix* sp. | Isactis sp. | ATCC 29345 |
|  |  | *Calothrix* sp. |  | ATCC 29199, ATCC 291 11, ATCC 29158, ATCC 291 12 |
| VI. Stigonematales (9 strains, 2 genera) | *Fischerella* | *Fischerella* sp. | *Mastigocladus sp.* | ATCC 27929 |
|  |  | *Fischerella* sp. | *Mastigocladus laminosus* | ATCC 29161, ATCC 29537, ATCC 29538, ATCC 29539, ATCC 29540 |
|  |  | *Fischerella muscicola* |  | ATCC 29114 |
|  | *Chlorogloeopsis* | *Chlorogloeopsis fritschii* |  | ATCC 27193 |
|  |  | *Chlorogloeopsis* sp. |  | ATCC 27181 |

- **Annex 4. Classification of Nostocales cyanoprokaryotes within the bacterial classification system according to Castenholz (2001) . HTF: High Temperature Form . *= registered species that requires taxonomic review**

| **Section Order** | **Subsection** | **Genera** | **Species** | **Synonym** | **Strain** |
| --- | --- | --- | --- | --- | --- |
| IV. Nostocales (36 strains a 6 genera) | Subsection IV. I | *Anabaena* | *Anabena variabilis* | *Cylindrospermium* sp. | PC7108 |
|  |  |  | *Anabaena cylindrica* |  | PCC 7122 |
|  |  |  | *Anabaena flos-aque* | *Aphanizomenon flos-aquae* | PC9302, PC9332 |
|  |  | *Anabaenopsis* | *Anabaenopsis* sp. | | PC9125 |
|  |  | *Aphanizomenon* | *Anabaenopsis elenkinii* |  | PC9420 |
|  |  |  | *Aphanizomenon flos-aquae* |  | PC7905 |
|  |  | *Cyanospira* | *Cyanospira rippkae* |  | Mag II 702 |
|  |  |  | *Cyanospira capsulata* |  | Mag I 504 |
|  |  |  | *Cyanospira* sp. |  | PC9501 |
|  |  | *Cilindrospermiopsis* | *Cilindrospermiopsis raciborskii* |  | AWT 205 |
|  |  | *Cylindrospermium* | *Cylindrospermium stagnale* |  | ATCC 29535 PCC 73 10 1. |
|  |  |  | *Cylindrospermium majus* |  | ATCC 33001 PCC 7604. |
|  |  | *Nodularia* | *Nodularia spumigena* |  | PCC 73104 |
|  |  |  | *Nodulara harveyana* |  | PCC 7804 |
|  |  | *Nostoc* | *Nostoc puntiforme* |  | PCC 73102 |
|  |  |  | *Nostoc* sp. (Cluster 3) |  | PCC 6705 |
|  |  |  | *Nostoc* sp. (Cluster 1) |  | PCC 7107 |
|  |  |  | *Nostoc* sp. (Cluster 2) | *Anabaenopsis circularis* | ATCC 27895 |
|  |  |  | *Nostoc* sp. (Cluster 4) |  | PCC 7524 |
|  |  |  | *Nostoc* sp. (Cluster 5) |  | PCC 6314 |
|  |  | *Scytonema* | *Scytonema hofmanni* |  | PCC 7110. |
|  | Subsection IV. II | *Calothrix* | *Calothrix* sp. (cluster 1) |  | PCC 7709 |
|  |  |  | *Calothrix desertica* |  | PCC 7102 |
|  |  |  | *Calothrix marchica* |  | PCC 7714 |
|  |  |  | *Calothrix parietina* |  | PCC 6303 |
|  |  |  | *Calothrix* sp. (cluster 2) | | PCC 7507 |
|  |  | *Rivularia* | *Rivularia* sp. | | PCC 7116 |
|  |  | *Tolypothrix* | *Tolypothrix* sp. | | PCC 7504 |
|  |  |  | *Tolypothrix tenuis* | | PCC 7101 |
|  |  |  | *Tolypothrix* (cluster 1) | *Calothrix membranacea* | PCC 7708 |
|  |  |  | *Tolypothrix* (cluster 2) | *Calotrhix* sp. | PCC 7415 |
|  |  | *Microchaete** | *Microchaete tenera* * |  | PCC 7126 |
| VI. Stigonematales (9 strains, 2 genera) |  | *Fischerella* | *Fischerella* sp. | *Mastigocladus sp.* |  |
|  |  |  | *Fischerella* sp. | *Mastigocladus laminosus* | PCC 7115 |
|  |  |  | *Hapalosiphon laminosus* | |  |
|  |  |  | *Mastigocladus* | |  |
|  |  | *Chlorogloeopsis* | *Chlorogloeopsis fritschii* | *Chlorogloea fritschii* | PCC 6912 |
|  |  |  | HFT *Mastigocladus* | |  |
|  |  |  | HFT *Chlorogloepsis* | |  |
|  |  | *Geitleria* | *Geitleria calcarea* | |  |
|  |  |  | *Scytonema julianum* | |  |
|  |  | *Iyengariella* | *Iyengariella endolithica* | |  |
|  |  | *Nostochopsis* | *Nostochopsis* sp. | |  |
|  |  | *Stigonema* | *Stigonema* cf. *ocellatum* | |  |
|  |  |  | *Stigonema*. cf. *minutum* | |  |
|  |  | *Mastigocladopsis* | *Mastigocladopsis* sp. |  |  |
|  |  | *Westellia* | *Westellia* sp. |  |  |
|  |  | *Doliocatella* | *Doliocatella* sp. |  |  |

- **• Annex 5. Nostocales according to Hoffmann *et al.* 2005. Bold: genera whose position is supported by molecular analysis**

| **Subclass** | **Order** | **Family** | **Genera** |
| --- | --- | --- | --- |
| **Nostochophycidae** | ***Nostocales*** | Scytonemataceae | ***Scytonema, Scytonematopsis****, Kyrtuthrix* |
|  |  | Symphonemataceae | *Symphonem*a orgnaismos with true Y-branching. Before Stigonematales. *Adrianema, Brachytrichia,* ***Herpyzonema****, Iyengariella,* ***Mastigocladopsis****, Parenchymorpha,* ***Symphyonemopsis, Symphyonema, Umezakia****, Voukiella* |
|  |  | Borzinemataceae | *Borzinema, Handeliella, Schmidleinema, Seguenzaea, Spelaeopogon* |
|  |  | Rivulariaceae | ***Rivularia, Calothrix, Gloeothrichia****, Dichothrix, Gardnerula, Gloeotrichia, Isactis?, Rivularia, Sacconema)* |
|  |  | Microchaetaceae | *Microchaete Spiritiensis Tolypothrix* |
|  |  | Nostocaceae | ***Nostoc, Anabaena, Trichormus, Anabaenopsis, Aphanizomenon, Cylindrospermiopsis, Nodularia****.* Generally planktonic |
|  |  | Chlorogoeopsidaceae | ***Chlorogoeopsis*** |
|  |  | Hapalosiphonaceae | ***Hapalosiphon, Mastigocladus*** with true T-branching. Before Stigonematales |
|  |  | Loriellaceae | *Loriella, Geitleria* |
|  |  | Stigonemataceae | ***Stigonema, Capsopsira*** with true branching. Before Stigonematales |

- **Annex 6. Classification System proposed in Komárek (2013) . In bold and blue = species with molecular information and type specimen . In (bold) and purple = species with untyped molecular information . In italics and yellow color = species without phylogenetic support . In (italics) and green color = species with taxonomic problems or invalid . With “name” symbol in red rectangle = the nature and status of the species is not clear**

| **Order** | **Family** | **Génera** |  |
| --- | --- | --- | --- |
| **Nostocales** | **Scytonemataceae** | ***Brasilonema Fiore,*** Sant-Anna, de Paiva Azevedo, Komárek, Kastovsky, Sulek & Lorenzi, 2007 |  |
|  |  | ***Chakia*** *E.* Zapomelová & J.Komárek, 2013 |  |
|  |  | *Kyrtuthrix* Ercegovic, 1929 |  |
|  |  | *Petalonema* M.J.Berkeley ex Wolle, 1887 |  |
|  |  | ***(Scytonema)*** C.Agardh ex É.Bornet & C.Flahault, 1886 |  |
|  |  | ***(Scytonematopsis)*** Kisseleva, 1930 |  |
|  |  | *(Scytonematopsis* secc. *Myocrotes)* É.Bornet & C.Flahault, 1886 |  |
|  | **Symphonemataceae** | *Adrianema* G.De Toni, 1936, nom. illeg. |  |
|  |  | *Brachytrichia* Zanardini ex Bornet & Flahault, 1886 |  |
|  |  | ***(Herpyzonema)*** Weber Bosse, 1913 |  |
|  |  | ***Ifinoe*** Lamprinou & Pantazidou 2011 |  |
|  |  | *Iyengariella* Desikachary, 1953 |  |
|  |  | ***Loriellopsis*** Hernández-Mariné & Canals, 2011 |  |
|  |  | ***(Mastigocladopsis)*** M.O.P.Iyengar & T.V.Desikachary, 1946 |  |
|  |  | *Parenchymorpha* C.K.Tseng & M.Hua, 1984 |  |
|  |  | ***Symphyonema*** C.-C.Jao, 1944 |  |
|  |  | ***Symphyonemopsis*** G.L.Tiwari & A.K.Mitra 1969 |  |
|  | **Rivulariaceae** | ***(Calothrix)*** C.Agardh ex Bornet & Flahault, 1886 |  |
|  |  | *Dichothrix* Zanardini ex Bornet & Flahault, 1886 |  |
|  |  | *Gardnerula* G.De Toni, 1936 |  |
|  |  | ***(Gloeotrichia)*** J.Agardh ex Bornet & Flahault, 1886 |  |
|  |  | *Isactis W*olle 1887 |  |
|  |  | ***(Rivularia)*** C.Agardh ex Bornet & Flahault, 1886, nom. cons. |  |
|  |  | *Sacconema* Borzì ex Bornet & Flahault, 1886 |  |
|  | **Microchaetaceae.** Including Borzinemataceae | *Borzinema (Borzì) G.De Toni 1936* |  |
|  |  | ***Calochaete*** Hauer, Bohunická & Mühlsteinová, 2013 |  |
|  |  | *Camptylonemopsis* Desikachary, 1948 |  |
|  |  | ***Coleodesmium*** A.Borzì ex L.Geitler, 1942 incluye *Coleodesmiopsis* Dutt, Datta & Gupta, 1982 |  |
|  |  | ***Dactylothamnos Fiore,*** Genuario & Komárek *et al.* 2015 |  |
|  |  | *Fortiea G.De Toni, 1936* |  |
|  |  | ***Godleya*** P.M.Novis & G.Visnovsky, 2011 |  |
|  |  | ***(Hassallia)*** Trevisan, 1848 |  |
|  |  | ***(Microchaete)*** Thuret ex Bornet & Flahault, 1886, nom. cons. |  |
|  |  | *Ophiothrix* Sant'anna, T.M.P.Azevedo, J.Kastovský & J.Komárek, 2010, nom. illeg. |  |
|  |  | ***Rexia*** D.A.Casamatta, S.R.Gomez & J.R.Johansen, 2006 |  |
|  |  | *Seguenzaea* Borzì, 1907 |  |
|  |  | ***Spirirestis*** V.R.Flechtner & J.R.Johansen, 2002 |  |
|  |  | ***(Streptostemon)*** Sant'Anna, Azevedo, Kaštovský & Komárek, 2010 |  |
|  |  | ***(Tolypothrix)*** Kützing ex Bornet & Flahault, 1886 |  |
|  |  | ***Toxopsis*** Lamprinou & al., 2012 |  |
|  | **Chlorogloeopsidaceae** | ***Chlorogloeopsis*** Maithy, 1975, nom. illeg. |  |
|  | **Hapalosiphonaceae.**  Including Mastogocladaceae | *Albrightia* J.J.Copeland, 1936 |  |
|  |  | *Brachytrichiopsis* C.-C.Jao, 1944 |  |
|  |  | *Chondrogloea* W.Schmidle, 1901 |  |
|  |  | *Colteronema* J.J.Copeland, 1936 |  |
|  |  | ***Geitleria***  I.Friedmann, 1955 |  |
|  |  | ***(Hapalosiphon)*** Nägeli ex Bornet & Flahault, 1886 |  |
|  |  | *Loefgrenia* Gomont, 1896 |  |
|  |  | *Loriella* Borzì, 1892 |  |
|  |  | *Mastigocoleopsis* Geitler, 1925 |  |
|  |  | ***Mastigocladus*** Cohn ex O.Kirchner, 1898 |  |
|  |  | *Matteia* Borzì, 1907 |  |
|  |  | *Thalpophila* Borzì, 1907 |  |
|  |  | ***(Westiella)*** Borzì, 1907 |  |
|  | **Nostochopsidaceae** | *?Baradlaia* P.Palik, 1960 |  |
|  |  | *Mastigocoleus* Lagerheim ex Bornet & Flahault, 1886 |  |
|  |  | ***Nostochopsis*** H.C.Wood ex Bornet & Flahault, 1886 |  |
|  | **Fischerellaceae** | ***(Fischerella)*** (Bornet & Flahault) Gomont, 1895 |  |
|  |  | *Fischerellopsis* F.E.Fritsch, 1932 |  |
|  |  | *Handeliella* Skuja, 1937 |  |
|  |  | *Hyphomorpha* Borzì, 1916 |  |
|  |  | *Leptopogon* A.Borzì, 1907 |  |
|  |  | *Letestuinema* Frémy, 1930 |  |
|  |  | *Schmidleinema* G.De Toni, 1936 |  |
|  |  | *Spelaeopogon* Borzì, 1907 |  |
|  |  | ***Westiellopsis*** Janet, 1941. ***Incluye*** Parthasarathiella Subba Raju 1962 |  |
|  | **Stigonemataceae** Including: Stigonemataceae, Capsopsiraceae, Pulvinulariaceae | ***(Capsopsira)*** Kützing ex Bornet & Flahault, 1886 |  |
|  |  | *Cyanobotrys* Hoffmann, 1991 |  |
|  |  | *Desmosiphon* Borzì, 1907 |  |
|  |  | *Doliocatella* L.Geitler, 1933 |  |
|  |  | *Homoeoptyche* Skuja, 1944 |  |
|  |  | *Nematoplaca* L.Geitler, 1933 |  |
|  |  | *Pulvinularia* Borzì, 1916 |  |
|  |  | *Stauromatonema* Frémy, 1930 |  |
|  |  | ***(Stigonema)*** C.Agardh ex Bornet & Flahault, 1886 |  |
|  | **Nostocaceae** | ***Anabaena*** Bory ex Bornet & Flahault, 1886, nom. cons. |  |
|  |  | ***Anabaena/Aphanizomenon*** |  |
|  |  | ***(Anabena) Cluster B*** |  |
|  |  | ***Anabaenopsis*** V.V.Miller, 1923 |  |
|  |  | ***Aphanizomenon*** Morren ex Bornet & Flahault, 1886 '1888' |  |
|  |  | ***(Aulosira)*** O.Kirchner ex Bornet & Flahault, 1886 |  |
|  |  | ***Chrysosporum*** E.Zapomelová, O.Skaácelová, P.Pumann, R.Kopp & E.Janecek, 2012 |  |
|  |  | ***Cronbergia*** J.Komárek, E.Zapomelová & F.Hindák, 2010 |  |
|  |  | ***Cuspidothrix*** Rajaniemi & al., 2005 |  |
|  |  | ***Cylindrospermopsis*** G.Seenayya & N.Subba Raju, 1972 |  |
|  |  | ***(Cylindrospermium)*** G.Seenayya & N.Subba Raju, 1972 |  |
|  |  | ***Cyanospira*** Chodat, 1921 |  |
|  |  | ***Desmonostoc*** Hrouzek & S.Ventura, 2013 |  |
|  |  | ***Dolichospermum*** (Ralfs ex Bornet & Flahault) P.Wacklin, L.Hoffmann & J.Komárek, 2009 |  |
|  |  | ***Hydrocoryne*** Schwabe ex Bornet & Flahault, 1886.  ***Include*** *Hormothamnion* |  |
|  |  | *?Isocystis* A.Borzì ex É.Bornet & C.Flahault, 1886 |  |
|  |  | ***Macrospermum*** Komarék, 2008 |  |
|  |  | ***Mojavia*** Reháková & J.R.Johansen, 2007 |  |
|  |  | ***Nodularia*** Mertens ex Bornet & Flahault, 1886, nom. cons. |  |
|  |  | ***Nostoc*** Vaucher ex Bornet & Flahault, 1886 |  |
|  |  | ***Raphidiopsis*** F.E.Fritsch & M.F.Rich, 1929 |  |
|  |  | ***Richelia*** J.Schmidt, 1901 |  |
|  |  | ***Sphaerospermopsis*** Zapomelová & al., 2010 |  |
|  |  | ***Trichormus*** (Ralfs ex Bornet & Flahault) Komárek & Anagnostidis, 1989 |  |
|  |  | ***Umezakia*** M.Watanabe, 1987 |  |
|  |  | ***Wollea*** Bornet & Flahault, 1886 |  |

- **Annex 7. Classification system proposed in Hauer & Komárek (2024), in the Cyano DB2 database.**

| **Order** | **Family** | **Genera** |  |
| --- | --- | --- | --- |
| **Nostocales** | **Aphanizomenonaceae** | *Amphiheterocytum* Sant Anna *et al.* 2019 | |
|  |  | *Anabaenopsis (*Woloszyńska) Miller 1923. | |
|  |  | *Aphanizomenon* Morren ex Bornet et Flahault 1888. | |
|  |  | *Cuspidothrix* Rajaniemi, Komárek, Willame, Hrouzek, Kaštovská, Hoffmann et Sivonen, 2005. | |
|  |  | *Cyanospira* Florenzano, Sili, Pelosi et Vincenzini 1985. | |
|  |  | *Cylindrospermopsis* Seenayya et Subba Raju, 1972. | |
|  |  | *Dolichospermum* (Ralfs ex Bornet et Flahault) Wacklin, Hoffmann et Komárek, 2009. | |
|  |  | *Chrysosporum* Zapomelova, Skácelová, Pumman, Kopp et Janeček, 2012. | |
|  |  | *Neocyanospir*a Molinari et Guiry 2021. | |
|  |  | *Nodularia* (Mertens in Jürgens) ex Bornet et Flahault 1888, | |
|  |  | *Raphidiopsis* Fritsch et Rich, 1929. | |
|  |  | *Sphaerospermopsi*s Zapomělová, Jezberová, Hrouzek, Hisem, Řeháková et Komárková, 2010. | |
|  | **Calotrichaceae** | *Calothrix* Agardh ex Bornet et Flahault, 1886 | |
|  |  | *Dulcicalothrix* Saraf, Sudkar, Dawda, Gaysina, Gabidullin, Kumat, Behere, Kotulkar, Batule et Singh 2019 | |
|  |  | *Fulbrightiella* N. Kumar, P. Singh et J.R. Johansen 2022. | |
|  |  | S*herwoodiella* J.R. Johansen et P. Singh 2022. | |
|  | **Capsosiraceae** | *Capsosira* Kützing ex Bornet et Flahault, 1888. | |
|  |  | *Desmosiphon* Borzi, 1907. | |
|  |  | *Hyphomorpha* Borzi, 1916. | |
|  |  | *Letestuinem*a Frémy, 1930 | |
|  |  | *Nematoplaca* Geitler, 1933 | |
|  |  | *Stauromatonema* Frémy, 1930. | |
|  | **Cyanomargaritaceae** | *Cyanomargarita* Shalygin, Shalygina et Johansen 2017*.* | |
|  | **Dapisostemonaceae** | *Dapisostemon* Hentschke, Sant’Anna et Johansen 2016. | |
|  | **Fortieaceae** | *Fortiea* De Toni 1936. | |
|  | **Geitleriaceae** | *Geitleria* Friedmann 1955. | |
|  |  | *Gloeotrichia* J. Agardh ex Bornet et Flahault 1886 | |
|  | **Godleyaceae** | *Godleya* Novis et Visnovsky 2011. | |
|  |  | T*oxopsis* Lamprinou, Skaraki, Kotoulas, Economou-Amilli et Pantazidou 2012. | |
|  | **Hapalosiphonaceae** | *Albrightia* Copeland 1936. | |
|  |  | *Baradlaia* Palik 1960. | |
|  |  | *Brachytrichiopsis* Jao 1944 | |
|  |  | *Colteronem*a Copeland 1936. | |
|  |  | *Dictyophoron* Komárek, Komárková, Ventura, Kozlíková-Zapomělová et Rejmánková 2017 | |
|  |  | *Fischerella* (Bornet et Flahault) Gomont, 1895 | |
|  |  | *Fischerellopsis* Fritsch 1932. | |
|  |  | *Handeliella* Skuja in Handel-Mazzetti, 1937 | |
|  |  | *Hapalosiphon* Nägeli in Kützing ex Bornet et Flahault 1887. | |
|  |  | *Chondrogloea* Schmidle 1901 | |
|  |  | *Leptopogon* Borzi, 1906. | |
|  |  | *Loefgrenia* Gomont 1896 | |
|  |  | *Loriella* Borzì 1892. | |
|  |  | *Mastigocladus* Cohn ex Kirchner 1898. | |
|  |  | *Mastigocoleopsis* Geitler 1925 | |
|  |  | *Mastigocoleus* Lagerheim ex Bornet et Flahault 1887 | |
|  |  | *Matteia* Borzì 1907. | |
|  |  | *Neowestioellopsis* Kabirnataj, Nemazadeh, Talebi, Tabatabaei et Singh 2018 | |
|  |  | *Nostochopsis* Wood ex Bornet et Flahault 1886. | |
|  |  | *Pelatocladus* Johansen et Vaccarino*.* 2016 | |
|  |  | *Reptodigitus* Casamatta, Villanueva, Stocks, Vaccarino et Johansen 2020 | |
|  |  | *Schmidleinema* De Toni, 1936 | |
|  |  | *Spelaeopogon* Borzi, 1907 | |
|  |  | *Thalpophila* Borzì 1907. | |
|  |  | *Westiella* Borzì 1907 | |
|  |  | *Westiellopsis* Janet, 1941 | |
|  | **Heteroscytonemataceae** | *Heteroscytonema* McGregor et Sendal 2018, |  |
|  | **Chlorogloeopsidaceae** | *Heterocyanococcus* Kufferath, 1929 |  |
|  |  | *Chlorogloeopsis* Mitra et Pandey 1967. | |
|  | **Microchaetaceae** | *Calochaete* Hauer, Bohunická & Mühlsteinová, | |
|  |  | *Camptylonemopsis* Desikachary 1948. | |
|  |  | *Microchaete* Thuret ex Bornet et Flahault 1887, nom. cons. | |
|  | **Nostocaceae** | *Aliinostoc* Bagchi, Bubey et Singh 2017. | |
|  |  | *Amazonocrinis* Alvarenga *et al.* 2021 | |
|  |  | Anabaena Bory ex Bornet et Flahault 1888. | |
|  |  | *Atlanticothrix* Alvarenga *et al.* 2021 | |
|  |  | *Aulosira* Kirchner ex Bornet et Flahault 1888 | |
|  |  | *Compactonostoc* F. Cai et R. Li 2019 | |
|  |  | *Constrictifilum* Chavadar *et al.* 2021 | |
|  |  | *Cronbergia* Komárek, Zapomělová et Hindák, 2010 | |
|  |  | *Cyanocohniella* Kaštovský, Berrendero, Hladil et Johansen 2014 | |
|  |  | *Cylindrospermum* Kützing ex Bornet et Flahault 1888 | |
|  |  | *Dendronalium* Alvarenga *et al.* 2021 | |
|  |  | *Desikacharya* Saraf, Dawda et P. Singh 2019. | |
|  |  | *Desmonostoc* Hrouzek et Ventura, 2013 | |
|  |  | *Goleter* Miscoe et Johansen 2016 | |
|  |  | *Halotia* Genuarion *et al.*, 2015 | |
|  |  | *Hormothamnion* Grunow ex Bornet et Flahault, 1888 | |
|  |  | *Hydrocoryne* Schwabe ex Bornet et Flahault 1888 | |
|  |  | *Isocystis* Borzì ex Bornet et Flahault 1888 | |
|  |  | *Johanseniella* Pal et Singh 2022 | |
|  |  | *Komarekiella* G.S. Hentschke, J.R. Johansen et C.L. Sant’anna 2017. | |
|  |  | *Macrospermum* Komárek, 2008 | |
|  |  | *Minunostoc* F. Cai et R. Li 2019. | |
|  |  | *Mojavia* Řeháková et Johansen 2007 | |
|  |  | *Neowollea* Tawong 2019 | |
|  |  | *Nostoc* Vaucher ex Bornet et Flahault, 1888 | |
|  |  | *Parakomarekiella* Soares, Ramos et Portugal 2020. | |
|  |  | *Pseudoaliinostoc* Lee, Ki et Lee 2021 | |
|  |  | *Purpureonostoc* Cai et Li 2020 | |
|  |  | *Richelia* J. Schmidt in Ostenfeld et J. Schmidt 1901 | |
|  |  | *Roholtiella* Bohunická, Pietrasiak et Johansen 2015 | |
|  |  | *Thiochaete* Welsh 1961 | |
|  |  | *Trichormus* (Ralfs ex Bornet et Flahault) Komárek et Anagnostidis 1989 | |
|  |  | *Violetonostoc* F. Cai et R. Li 2020 | |
|  |  | *Wollea* Bornet et Flahault 1888. | |
|  | **Rivulariaceae** | *Amphithrix* Bornet et Flahault, 1886 | |
|  |  | *Dichothrix* Zanardini ex Bornet et Flahault 1886 | |
|  |  | *Gardnerula* De Toni, 1936 | |
|  |  | *Isactis* Thuret ex Bornet et Flahault 1886 | |
|  |  | *Kyrtuthrix* Ercegović 1929. | |
|  |  | *Macrochaete* Berrendero, Johansen et Kastovsky 2016 | |
|  |  | *Nunduva* González–Resendiz, León–Tejera et Johansen 2013 | |
|  |  | *Phylonema* Alvarenga *et al.* 2016 | |
|  |  | *Physactis* Küzing 1843 | |
|  |  | *Rivularia* [Roth] C. Agardh ex Bornet et Flahault 1886 | |
|  |  | *Sacconema* Borzi ex Bornet et Flahault, 1886 | |
|  | **Scytonemataceae** | *Aetokthonos* Wilde et Johansen 2014, | |
|  |  | *Brasilonema* Fiore, Sant'Anna, Azevedo, Komárek, Kaštovský, Sulek et Lorenzi, 2007. | |
|  |  | *Cyanopiothrix* Molinari et Guiry 2021. | |
|  |  | *Ewamiania* McGregor & Sendall 2017 | |
|  |  | *Chakia* Komárková, Zapomělová et Komárek, 2013. | |
|  |  | *Iningainema* McGregor et Sendall 2017. | |
|  |  | *Iphinoe* Lamprinou et Pantazidou, 2011 | |
|  |  | *Petalonema* Berkeley ex Correns, 1889. | |
|  |  | *Scytonema* Agardh ex Bornet et Flahault 1887. | |
|  |  | *Scytonematopsis* Kiseleva 1930 | |
|  |  | *Spelaeonaias* Lamprinou, Christodoulou, Hernández -Mariné et Economou-Amilli in Lamprinou *et al.* 2016 | |
|  | **Stigonemataceae** | *Caeruleovitis* Molinari & Sánchez Ocharan 202 | |
|  |  | *Cyanobotrys* L. Hoffman, 1991. | |
|  |  | *Doliocatella* Geitler 1933 | |
|  |  | *Homoeoptyche* Skuja, 1944. | |
|  |  | Pulvinularia Borzi, 1916 | |
|  |  | *Stigonema* Agardh ex Bornet et Flahault, 1888 | |
|  | **Symphyonemataceae** | *Adrianema* De Toni 1936 | |
|  |  | *Brachytrichia* Zanardini ex Bornet et Flahault 1887 | |
|  |  | *Herpyzonema* Weber van Bosse, 1913 | |
|  |  | *Iphianassa* Panou et Gkelis 2022 | |
|  |  | *Iyengariella* Desikachary 1953 | |
|  |  | *Loriellopsis* Hernández-Mariné et Canals, 2011 | |
|  |  | *Mastigocladopsis* Iyengar et Desikachary, 1946 | |
|  |  | *Parenchymorpha* Tseng et Hua 1984 | |
|  |  | *Symphyonema* Jao, 1944 | |
|  |  | Symphyonemopsis Tiwari et Mitra, 1969. | |
|  |  | *Voukiella* Ercegović, 1925 | |
|  | **Tolypotrichaceae** | *Borzinema* De Toni 1936 | |
|  |  | *Coleodesmium* Borzì ex Geitler 1942 | |
|  |  | *Croatella* Ercegović 1925. | |
|  |  | *Dactylothamnos* Komárek, Genuário, Fiore et Elster 2015. | |
|  |  | *Hassallia* Berkeley ex Bornet et Flahault 1888 | |
|  |  | *Kryptousia* Alvarenga, Andreote, Branco et Fiore | |
|  |  | *Rexia* Casamatta, Gomez et Johansen 2006 | |
|  |  | *Seguenzaea* Borzì 1907 | |
|  |  | *Spirirestis* Flechtner et Johansen 2002 | |
|  |  | *Streptostemon* Sant Anna, Azevedo, Kaštovský et Komárek 2010 | |
|  |  | *Tolypothrix* Kützing ex Bornet et Flahault 1888 | |

**Annex 8. Classification System proposed by Komárek *et al.* (2013). In blue= Monophyletic species.**

| **Order** | **Family** | **Genera** | **Extra data** |
| --- | --- | --- | --- |
| **Nostocales** | **Scytonemataceae** | *Scytonema* | Monophyletic |
|  |  | *Brasilonema* | Monophyletic |
|  |  | *Scytonema* sect. Myochrotes | Monophyletic |
|  |  | *Chakia* | Monophyletic |
|  |  | *Petalonema* | Monophyletic |
|  |  | *Scytonematopsi* | Polyphyletic |
|  |  | *Ophiothrix* | Without secuences |
|  |  | *Kyrtuthrix* | Without secuences |
|  | **Symphyonemataceae** | *Adrianema* G.De Toni, 1936, nom. illeg. |  |
|  |  | *Brachytrichia* Zanardini ex Bornet et Flahault 1887 |  |
|  |  | *Herpyzonema Weber* van Bosse 1913 |  |
|  |  | *Ifinoe* Lamprinou et Pantazidou 2011 | Probably near of *Brasilonema* |
|  |  | *Iyengariella* Desikachary 1953 |  |
|  |  | *Loriellopsis* Hernandéz Mariné et Canals 2011 |  |
|  |  | *Parenchymorpha* Tseng et Hua 1984 |  |
|  |  | *Mastigocladopsis* Iyengar et Desikachary 1946 | Monophyletic |
|  |  | *Symphyonema* Jao 1944 | Monophyletic |
|  |  | *Voukiella* Ercegović 1925 |  |
|  |  | *Symphyonemopsis* Gugger et Hoffmann 2004 | Probably near of *Brasilonema* |
|  | **Rivulariaceae** | *Calothrix* Agardh ex Bornet et Flahault 1886 |  |
|  |  | *Dichothrix* Zanardini ex Bornet et Flahault 1886 |  |
|  |  | *Rivularia* C.Agardh ex Bornet & Flahault, 1886, nom. cons. |  |
|  |  | *Microchaete* Thuret ex Bornet & Flahault, 1886, nom. cons. | Probably belong here |
|  |  | *Gloeotrichia* Thuret ex Bornet & Flahault 1886 | Probably Nostocaceae |
|  |  | *Gardnerula* DeToni 1936 |  |
|  |  | *Isactis* Thuret ex Bornet et Flahault 1886 |  |
|  |  | *Sacconema* Borzě ex Bornet et Flahault 1886 |  |
|  | **Tolypothrichaceae** | *Borzinema* DeToni 1936 |  |
|  |  | *Coleodesmium* Borzě ex Geitler 1942 |  |
|  |  | *Dactylothamnos* Fiore *et al.* 2013 | Probably Scytonemataceae |
|  |  | *Hassallia* Berkeley ex Bornet et Flahault 1888 |  |
|  |  | *Rexia* Casamatta *et al.* 2006 |  |
|  |  | *Seguenzaea* Borzě 1907 |  |
|  |  | *Spirirestis* Flechtner et Johansen 2002 | Probably  Scytonemataceae |
|  |  | *Streptostemon* Sant’Anna *et al.* 2010 4 |  |
|  |  | *Tolypothrix* Kützing ex Bornet et Flahault 1887 |  |
|  | **Godleyaceae** | *Godleya* Novis et Visnovsky 2011 | Before microchaetaceae |
|  |  | *Toxopsis* Lamprinou *et al.* 2012 | Before microchaetaceae |
|  | **Chlorogloeopsidaceae Forming a monophyletic clade with a sister clade Hapalosiphonaceae** | *Chlorogloeopsis* Mitra et Pandey 1967 |  |
|  | **Hapalosiphonaceae** | *Fischerella* (Bornet et Flahault) Gomont 1895 | Monophyletic |
|  |  | *Mastigocladus* Cohn ex Kirchner 1898 | Monophyletic |
|  |  | *Westiellopsis* Janet 1941 | Monophyletic |
|  |  | *Nostochopsis* Wood ex Bornet & Flahault 1886 | Monophyletic |
|  |  | *Hapalosiphon* Nägeli in Kützing ex Bornet et Flahault 1887 | Monophyletic |
|  |  | *Mastigocoleus* Lagerheim ex Bornet | Monophyletic |
|  |  | *Aetokthonos* Wilde *et al.* 2014 |  |
|  |  | *Albrightia* Copeland 1936 |  |
|  |  | *Baradlaia* Palik 1960 |  |
|  |  | *Brachytrichiopsis* Jao 1944 |  |
|  |  | *Chondrogloea* Schmidle 1902 |  |
|  |  | *Colteronema* Copeland 1936 |  |
|  |  | *Fischerellopsis* Fritsch 1932 |  |
|  |  | *Geitleria* Friedmann 1955 |  |
|  |  | *Handeliella* Skuja 1937 |  |
|  |  | *Hyphomorpha* Borzě 1916 |  |
|  |  | *Leptopogon* Borzě 1917 |  |
|  |  | *Letestuinema* Frémy 1930 |  |
|  |  | *Loefgrenia* Gomont 1896 |  |
|  |  | *Loriella* Borzě 1892 |  |
|  |  | *Mastigocoleopsis* Geitler 1925 |  |
|  |  | *Matteia* Borzě 1907 |  |
|  |  | *Schmidleinema* DeToni 1936 |  |
|  |  | *Spelaeopogon* Borzě 1917 |  |
|  |  | *Thalpophila* Borzě 1907 |  |
|  |  | *Westiella* Borzě 1907 |  |
|  |  | *Capsosira* Kützing ex Bornet et Flahault 1887 |  |
|  |  | *Desmosiphon* Borzě 1907 |  |
|  |  | *Nematoplaca* Geitler 1933 |  |
|  |  | *Stauromatonema* Frémy 1930 |  |
|  |  | *Stigonema* C. Agardh ex Bornet et Flahault 1886 | Monophyletic at the family level, polyphyletic within the family |
|  |  | *Cyanobotrys* Hoffmann 1991 4 |  |
|  |  | *Doliocatella* Geitler 1933 4 |  |
|  |  | *Homoeoptyche* Skuja 1944 4 |  |
|  |  | *Pulvinularia* Borzě 1916 4 |  |
|  |  | *Gloeotrichia* Thuret ex Bornet & Flahault 1886 |  |
|  |  | *Heliotrichum* | It is now recognized as a synonym for Oscillatoria. Taxon of special interest required analysis |
|  |  | *Calothrix* with akinetes | Probably Nostocaceae |
|  |  | *Aphanizomenon* Morren ex Bornet et Flahault 1888 |  |
|  |  | *Sphaerospermopsis* Zapomělová *et al.* 2010 , Chrysosporum and possibly also Cyanocohniella |  |
|  |  | *Umezakia* M. Watanabe 1987 |  |
|  |  | *Dolichospermum* (Ralfs) Wacklin *et al.* 2009 |  |
|  |  | *Cuspidothrix* Rajaniemi *et al.* 2005 |  |
|  |  | *Chrysosporum* Zapomělová *et al.* 2012 |  |
|  |  | *Cyanocohniella* Kastovský, Berrendero Gómez, Hladil & J.R.Johansen, 2014 |  |
|  |  | *Nodularia* Mertens in Jürgens ex Bornet et Flahault 1888 | Monophyletic |
|  |  | *Raphidiopsis* Fritsch et Rich 1929 | Monophyletic |
|  |  | *Cylindrospermopsis* Seenayya et Subba Raju 1972 | Monophyletic |
|  |  | *Anabaenopsis* (Wołoszyńska) Miller 1923 1, 3 | Monophyletic |
|  |  | *Cyanospira* Florenzano *et al.* 1985 | Monophyletic |
|  | **Nostocaceae** | *Anabaena* Bory ex Bornet et Flahault 1886 | Polyphyletic |
|  |  | *Camptylonemopsis* Desikachary 1948 |  |
|  |  | *Coleospermopsis* Hauer *et al.* 2015 |  |
|  |  | *Cronbergia* Komárek *et al.* 2010 |  |
|  |  | *Cyanocohniella* Kaštovský *et al.* 2014 |  |
|  |  | *Cylindrospermum* Kützing ex Bornet et Flahault 1888 |  |
|  |  | *Desmonostoc* Hrouzek et Ventura 2013 | Monophyletic |
|  |  | *Goleter* Miscoe *et al.* 2015 |  |
|  |  | *Hydrocoryne* Schwabe ex Bornet et Flahault 1888 |  |
|  |  | *Isocystis* Borzě ex Bornet et Flahault 1888 |  |
|  |  | *Macrospermum* Komárek 2008 |  |
|  |  | *Mojavia* Řeháková et Johansen 2007 | Monophyletic |
|  |  | *Nostoc* Vaucher ex Bornet & Flahault 1888 | Polyphyletic |
|  |  | *Richelia* J. Schmidt in Ostenfeld et J. Schmidt 1901 |  |
|  |  | *Spelaea* Miscoe *et al.* 2013 |  |
|  |  | *Tolypothrichopsis* Hauer *et al.* 2015 |  |
|  |  | *Trichormus* (Ralfs ex Bornet et Flahault) Komárek et Anagnostidis 1989 | Polyphyletic |
|  |  | Wollea Bornet et Flahault 1888 | Polyphyletic |
|  |  | *Microchaete* Thuret ex Bornet & Flahault 1886 | Before *Fremyella*, now as *nom cons* |
|  | **Fortieaceae** | *Aulosira* Kirchner ex Bornet et Flahault 1886 |  |
|  |  | *Calochaete* Hauer *et al.* 2013 |  |
|  |  | *Coleospermum* Kirchner in Cohn 1878 |  |
|  |  | *Fortiea* De-Toni 1936 |  |
|  |  | *Roholtiella* Bohunická *et al.* 2015 |  |

Graphical abstract:

BLURB FOR ETOC:
In this manuscript we contribute with a revision of Cyanoprokaryotes Nostocales in the Gulf of Mexico and the Mexican Caribbean, the list of species was updated taxonomically. Also, the historical analysis of their taxonomy was carried out.
